# Sequence and trajectory of early Alzheimer’s disease-related tau inclusions in the hippocampal formation of cases without amyloid-β deposits

**DOI:** 10.1007/s00401-025-02862-x

**Published:** 2025-05-23

**Authors:** Heiko Braak, Benjamin Mayer, Simone Feldengut, Michael Schön, Kelly Del Tredici

**Affiliations:** 1 Department of Neurology, Center for Biomedical Research, Clinical Neuroanatomy, University of Ulm, Helmholtzstrasse 8/1, 89081 Ulm, Germany; 2 Institute of Epidemiology and Medical Biometry, University of Ulm, 89075 Ulm, Germany; 3 Institute for Anatomy and Cell Biology, University of Ulm, 89081 Ulm, Germany

**Keywords:** Alzheimer’s disease, AT8, Cornu ammonis, Dendritic tau, Entorhinal region, Fascia dentata, Ghost threads, Hippocampal formation, Neuronal loss, Neurofibrillary tangles/neuropil threads, Neuron-to-neuron transmission, Perinuclear rims, Pretangle, Thorny excrescences

## Abstract

Sporadic Alzheimer’s disease (AD) involves specific neuronal types and progresses in a systematic manner, permitting subdivision into six neuropathological stages. Neurofibrillary tangle (NFT) stages I–III display abnormal tau inclusions confined to subcortical nuclei and temporal allocortical regions, frequently without amyloid β (Aβ) deposition. We previously suggested a sequence of neuronal involvement in AD that could proceed from entorhinal pre-α cells to hippocampal prosubicular pyramidal cells and the CA1/CA2 sectors, from there to the thorny excrescences on mossy cells in CA3/CA4, and, finally, from the mossy cells to dentate fascia (Fd) granular cells. Here, we aimed to see if associations existed between the early NFT stages I–III, when Aβ deposits are frequently absent, and the following four categories: (1) anatomical regions and abnormal morphological tau changes in region-specific layers, (2) nerve cell loss, (3) APOE genotype, and (4) the trajectory (directionality) of tau progression in the hippocampal formation. To do so, we examined the transentorhinal/entorhinal regions and hippocampal formation using AT8-immunohistochemistry in 100 µm sections from *N* = 308 brains with tau inclusions lacking Aβ deposits between NFT stages I and III (average age at death 66.7 years for females, 66.4 years for males). Our results indicated a significantly (*p* < 0.001) ordered progression of abnormal tau in a direction opposite to currently known unidirectional intrahippocampal connections, thereby indirectly supporting the idea of transneuronal abnormal tau spreading, i.e., anterogradely, through the hippocampal formation. Tau-related neuronal loss was also significant (*p* < 0.001 for the transentorhinal/entorhinal regions and for sectors CA1/CA2 and *p* = 0.003 for CA3/CA4/Fd). These findings challenge the amyloid cascade and the PART hypotheses, corroborating the concept that early AD-related tau inclusions and tau-related neuronal loss occur independently of Aβ deposition.

**Supplementary Information:**

The online version contains supplementary material available at 10.1007/s00401-025-02862-x.

## Introduction

Sporadic Alzheimer’s disease (AD) is a chiefly homogeneous human tauopathy, which preferentially involves late-developing and late-maturing projection neurons of the central nervous system that generate a long and late-myelinating axon [3, 4, 23, 43, 100, 104, 106, 112]. The pathological process begins early in life without extracellular amyloid β (Aβ) deposition [21, 27].

AD-related brain changes include the abnormal and AT8-immunopositive tau protein in the form of partially soluble and non-fibrillar pretangles that subsequently form argyrophilic fibrils and then neuropil threads in dendrites (NTs), and neurofibrillary tangles (NFTs) in cell somata [14, 15, 110, 120]. By contrast, abnormal tau in axons usually resists conversion into insoluble and fibrillar tau [23]. Axonal seeds of pretangle tau are thought to transfer the pathology anterogradely from involved nerve cells to uninvolved neurons through axonal connectivities [30, 45, 58, 59, 77, 81, 82].

The predictable regional pattern of the tau inclusions in the brain permits the distinction of neuropathological stages [2, 5, 18, 23, 26, 27, 67, 116]. Initially, cortical tau changes remain virtually confined to the medial temporal lobe and, notably, they usually develop in the absence of Aβ deposition. The abnormal tau inclusions are invariant characteristics of the early AD-related process and are necessary for the development of the following disease stages that ultimately result in clinically detectable phases of AD [5, 27, 116].

**Fig. 1:**
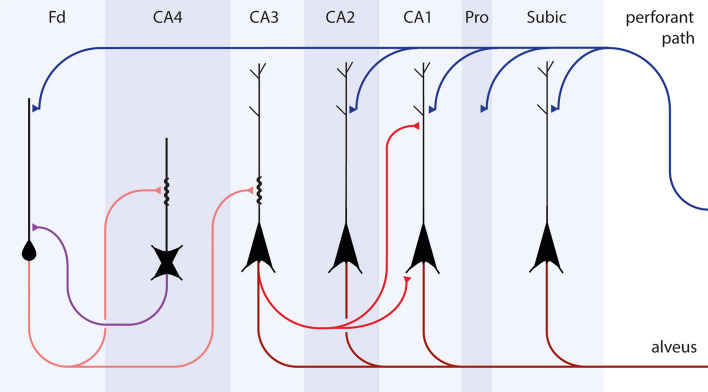
Summary diagram of allocortical connectivities of the temporal lobe. Outer layers of the entorhinal and transentorhinal regions project via the perforant path (*blue connections*) to the Ammon’s horn and predominantly to the dentate fascia (Fd). Granule cells of the dentate fascia project via mossy fibers to excrescences of multipolar cells in CA4 and pyramidal cells in CA3 (*light red connections*). Axons of CA3 pyramidal cells give off Schaffer collaterals to dendrites of CA1 pyramidal cells in the stratum lacunosum and stratum oriens (*dark red connections*). CA1 pyramidal cells project via the ammonic-subicular pathway (not shown) to the subiculum, and subicular efferents reach the deep layer pri-α of the entorhinal region and the alveus (*dark red connections*)

Known anatomical connectivities within the temporal allocortex include portions of the perforant path that are generated from the outer layers of the entorhinal region and head towards the dentate fascia (Fd) [6] (Fig. 1, blue connections). From there, axons of dentate granular cells (mossy fibers) contact projection neurons of CA4/CA3 (Fig. 1, light red). Schaffer collaterals connect to CA1 (Fig. 1, dark red), and the ammonic-subicular pathway finally reaches the output region, the subiculum [70] (Suppl. Fig. 1). However, in a previous study, we suggested a sequence of neuronal involvement in AD that could proceed in the opposite direction, namely, from the entorhinal region to the hippocampal prosubiculum and CA1/CA2 sectors, then to the thorny excrescences and mossy cells in CA3/CA4, whence it reaches the dentate granular cells [25].

Here, we present a hypothetical route of potential tau spreading through allocortical portions of the temporal lobe and point to its importance for the early AD pathological process using AT8-immunostained sections from the transentorhinal/entorhinal regions and hippocampal formation from *N* = 308 autopsy brains. We asked if associations existed between the early NFT stages I–III when Aβ deposits are absent and the following four categories: (**1**) anatomical regions and local layers (laminae) with abnormal morphological changes, (**2**) nerve cell loss, (**3**) APOE genotype, and (**4**) the here-proposed directionality (trajectory) of tau progression in the hippocampal formation.

## Material and methods

### Study cohort

This retrospective study was conducted in compliance with ethical principles originating in, or derived from, the latest version of the Declaration of Helsinki, Ulm University ethics committee guidelines, and with German federal and state law governing human tissue usage. Informed written permission was obtained from all patients and/or their next of kin for autopsy.

We included brains from *N* = 308 cognitively unimpaired individuals (CDR 0, CERAD 0 based on clinical and/or postmortem chart reviews in our University of Ulm brain bank and tissue database) of European ancestry neuropathologically staged according to previously published protocols [2, 26, 27, 67]. All 308 cases fit the description for definite PART (primary age-related tauopathy), i.e., cognitively unimpaired individuals (here, regardless of age) with NFT/NT stages I-VI, Aβ phase 0, and CERAD none [36, 50].

**Fig. 2:**
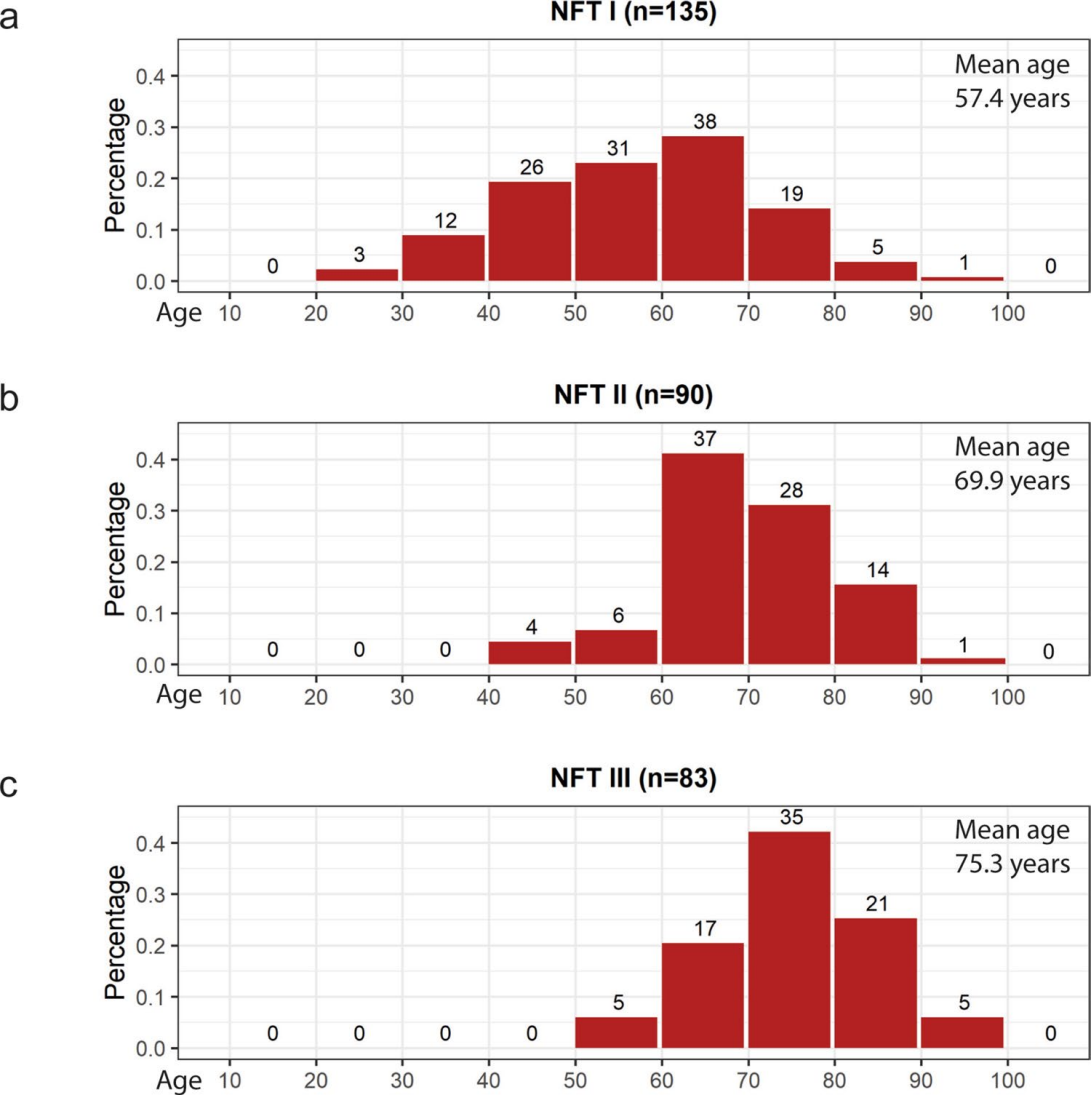
Abnormal intraneuronal tau deposits in the absence of amyloid-β in *N* = 308 autopsy cases according to decades (ages of cohort 28–100 years). The distribution of age groups is shown within each NFT subgroup (stages I–III). Columns in (**a**–**c**) display the frequency of cases in relation to the total number of cases in the respective stage. The graphs show the prevalences of AD-related non-argyrophilic AT8-positive pretangles and argyrophilic neurofibrillary changes in NFT stages I (**a**), II (**b**), and III (**c**). The cases show a Gaussian distribution. The mean age for cases in NFT stage I was 57.4 years, for NFT stage II 69.9 years, and for NFT III cases 75.3 years, i.e., the average age increased with disease progression. We computed a Monte Carlo *p*-value (250,000 replications) for the null hypothesis that the sample data come from a normal distribution. No significant skewness existed in either NFT class (*p* = 0.705 for NFT I, *p* = 0.864 for NFT II, and *p* = 0.609 for NFT III)

Brains with neuropathologically confirmed synucleinopathies or TDP-43 frontotemporal lobar dementia were excluded. Additional exclusionary criteria were the presence of Aβ deposition, hippocampal sclerosis, cerebral bleedings, epilepsy, ischemic stroke, cerebrovascular disease, or tauopathies other than AD, e.g., argyrophilic grain disease (AGD), progressive supranuclear palsy (PSP), corticobasal degeneration (CBD), Pick’s disease (PiD), or chronic traumatic encephalopathy (CTE). Neuropathologic diagnoses for all excluded non-AD tauopathies had been made previously using 100 µm free-floating tissue sections and published protocols [16, 17, 39, 40, 64, 79, 89]. Demographic and neuropathological staging data for all cases (120 females, 188 males, age range 28–100 years; mean age 65.9 years) are summarized in Table 1. NFT stages I–III according to decades (age distribution) for all cases are shown in Fig. 2a–c.

**Table 1:** Early tau pathology in the absence of amyloid β deposits in N = 308 cases

Age group (yrs)	NFT I	NFT II	NFT III	Total
0–9	0	0	0	0
10–19	0	0	0	0
20–29	3 (2f/1 m) 2.22%	0	0	3 (2f/1 m) 1%
30–39	12 (4f/8 m) 8.90%	0	0	12 (4f/8 m) 4%
40–49	26 (11f/15 m) 19.26%	4 (3f/1 m) 4.44%	0	30 (14f/16 m) 10%
50–59	31 (11f/20 m) 22.96%	6 (1f/5 m) 6.67%	5 (1f/4 m) 6.02%	42 (13f/29 m) 14%
60–69	38 (14f/24 m) 28.15%	37 (11f/26 m) 41.11%	17 (9f/8 m) 20.49%	92 (34f/58 m) 30%
70–79	19 (7f/12 m) 14.07%	28 (12f/16 m) 31.11%	35 (17f/18 m) 42.17%	82 (36f/46 m) 27%
80–89	5 (1f/4 m) 3.70%	14 (7f/7 m) 15.56%	21 (8f/13 m) 25.30%	40 (16f/24 m) 12%
90–100	1 (0f/1 m) 0.74%	1 (0f/1 m) 1.11%	5 (1f/4 m) 6.02%	7 (1f/6 m) 2%
	**135 (50f/85 m)** **44%**	**90 (34f/56 m)** **29%**	**83 (36f/47 m)** **27%**	**308 (120f/188 m)** **100%**
Mean age in years (SD)	57.4 (13.4)	69.9 (10.2)	75.3 (8.8)	

Total values for each column appear in bold

For each age group, the respective number of individuals in NFT stages I, II and III is presented. Frequency according to decades, including ratio between females (**f**, *n* = 120) and males (**m**, *n* = 188); percentages in NFT stages I to III reach within each stage a cumulative percentage of 100%; percentage in total column within entire sample; age in years

*SD* standard deviation

### APOE genotyping

APOE status was available for 188 of 308 (61%) cases in our study cohort and included 79 females, and 109 males; age range 28–100. As in prior studies, the single largest group consisted of ε3/ε3 carriers [48, 97]. The breakdown of all carriers according to genotype, gender, and NFT stage is shown in Tables 2a, 2b.

**Table 2a:** APOE genotyping according to gender in 188/308 cases

*APOE*	Female, *n* = 79 (42.0%)	Male, *n* = 109 (58.0%)	Total, 188 (100%)
ε2/ε2	1	1	2
ε2/ε3	16	23	39
ε2/ε4	22	28	50
ε3/ε3	23	40	63
ε3/ε4	16	15	31
ε4/ε4	1	2	3

APOE ε3/ε3 was the most common variant in our cohort, as reported previously for the general population [7]. A single APOE ε4 allele increases the risk of developing AD two- to four-fold [48], and persons with APOE ε4/ε4 have an eight-fold to 12-fold increased risk of doing so [48]. Here, as elsewhere, APOE ε2/ε2 was the least common variant, and individuals with this variant or with the ε2/ε3 variant presumably were more resistant to AD [48]

**Table 2b:** APOE genotyping according to NFT/NT stage (AT8-IHC) in 188/308 cases

NFT stage	ε2/ε2, *n* = 2 (1.0%)	ε2/ε3, *n* = 39 (20.8%)	ε2/ε4, *n* = 50 (26.6%)	ε3/ε3, *n* = 63 (33.5%)	ε3/ε4, *n* = 31 (16.5%)	ε4/ε4, n = 3 (1.6%)	Total, 188 (100%)
I	1 (50.0%)	17 (43.6%)	26 (52.0%)	32 (50.8%)	13 (41.9%)	1 (33.3%)	90 (47.9%)
II	1 (50.0%)	10 (25.6%)	11 (22.0%)	22 (34.9%)	4 (12.9%)	2 (66.7%)	50 (26.6%)
III		12 (30.8%)	13 (26.0%)	9 (14.3%)	14 (45.2%)		48 (25.5%)

A total of 90 (47.9%) cases were assigned to NFT stage I, 50 (26.6%) to stage II, and 48 (25.5%) to NFT stage III

*NFT* neurofibrillary tangle, *NT* neuropil threads, *IHC* immunohistochemistry

**Table 2c:** APOE genotype distribution in 188/308 cases

Genotype	Genotype frequency (%)
ε2/ε2	1.06
ε2/ε3	20.74
ε2/ε4	27
ε3/ε3	33.51
ε3/ε4	16.49
ε4/ε4	1.6
	100

Genotype	Number of cases
ε2/ε2	*n* = 2
ε2/ε3	*n* = 39
ε2/ε4	*n* = 50
ε3/ε3	*n* = 63
ε3/ε4	*n* = 31
ε4/ε4	*n* = 3
	*n* = 188

The percentages for our gentotypes ε2/ε2 (1.06%) and ε4/ε4 (1.6%) approximated those in the general population. The ε2/ε3 (20.74%) and ε2/ε4 (27%) genotype frequencies were elevated (overrepresented)

**Table 2d:** Allele frequencies in 188/308 cases

Allele	Allele frequency (%)
ε2	24.7
ε3	52.1
ε4	23.1

The allele frequencies for **ε**2 and ε4 were elevated (overrepresented); the ε3 frequency was low (underrepresented): The ε2 allele frequency was 24.7% instead of 8% in the general population. The ε4 allele frequency was 23.1% instead of 14% in the general population (although not, as in AD, ca. 30–40%). The ε3 frequency was 52.1% instead of 78% in the general population

**Table 2e:** Allele frequencies in 188/308 cases

Genotype Mean age (yrs)	
ε2/ε2	60
ε2/ε3	65.07
ε2/ε4	66.12
ε3/ε3	64.93
ε3/ε4	64.93
ε4/ε4	61.33

The mean ages for the various APOE genotypes hardly differed. In addition, when the cases were divided into those with and those without an ε4 allele, the mean age in both groups did not differ: 65.5 years of age (SD 12.8) for those with the ε4 allele versus 65 years of age (SD 15.3) for those without. A difference of 0.5 years exists, and the associated t-test result failed to reach significance (*p* = 0.810)

*yrs* years, *SD* standard deviation

### Genetic analysis

APOE genotyping was performed on archival formaldehyde-fixed brain samples (pons, cerebellum). The tissue was fixed for up to a month in an aqueous solution of formaldehyde. The QIAamp DNA Mini Kit (Qiagen, catalogue no. 51306, Hilden, Germany) and Taq PCR Master Mix Kit (Qiagen, catalogue no. 201443, Hilden, Germany) were used according to the manufacturer’s instructions. DNA amplification for all PCR products was evaluated by 3% agarose gel electrophoresis. Aliquots of the PCR-amplified products were digested with restriction enzyme *AlfIII* (New England BioLabs R0541S, Ipswich, MA; 10U/µl), *HaeII* (New England BioLabs R0107S; 20U/µl), and *HaeI* (New England BioLabs R0139S; 20U/µl). Incubation took place at 37 °C for 3 h, heat inactivation at 80 °C for 20 min. For genotype analysis, the small fragment-sized cleavage products of *AlfIII*, *HaeII*, and *HaeI* were electrophoresed through 18% polyacrylamide gels stained with ethidium bromide and visualized with ultraviolet illumination.

### Tissue fixation, embedding, and sectioning

Brain tissue was fixed by immersion in a 4% buffered aqueous solution of formaldehyde for ca. 10–20 days prior to dissection and neuropathological assessment. As described previously [2, 26], three transversally cut tissue blocks required for neuropathological diagnosis and evaluation of NFT stages of AD-related changes were excised: The first block was dissected at the mid-uncal level through medial portions of the temporal lobe and encompassed anterior portions of the hippocampal formation and the parahippocampal gyrus (entorhinal and transentorhinal regions), including the occipitotemporal gyrus and inferior temporal gyrus up to the superior temporal gyrus. The second block was cut through the temporal lobe at the level of the lateral geniculate body and included middle portions of the hippocampal formation that perfectly showed its various subdivisions. The third block was oriented perpendicular to the calcarine fissure and contained portions of the peristriate region, the parastriate and the striate areas. Some of the tissue blocks were cryosectioned, and the majority were embedded in polyethylene glycol (PEG 1000, Merck, Carl Roth Ltd, Karlsruhe, Germany) and sectioned at a thickness of 100 µm on a tetrander microtome (Jung, Heidelberg, Germany), as described previously [19, 47]. Compared to routine paraffin-embedded sections of 4–5 µm or cryosections of 5–15 µm thickness, this unconventional section thickness permits the optical superimposition of biological structures, including pretangles, NFT/NT, and Aβ deposits, and nerve cells with their dendritic arborizations, as well as large portions of the arborization around each involved neuron [19].

### Staining and immunocytochemistry

One set of free-floating sections from each case was processed according to an updated and simplified protocol for staining with aldehyde fuchsine to visualize the pigmentation properties of the different nerve cell populations in the adult human brain, as well as intraneuronal and extraneuronal lipofuscin pigment deposits; counterstaining with Darrow red was performed to visualize basophilic material in nerve cells [12, 19, 23]. Aldehyde fuchsine also visualizes cortical microinfarctions and CD86-immunopositive macrophages in brain tissue [28]. Silver methods (Campbell-Switzer, Gallyas silver staining) as well as 4G8 immunohistochemistry were used here to visualize Aβ deposits and argyrophilic neurofibrillary lesions [19, 23, 26, 32, 54, 67].

For immunohistochemistry, sets of free-floating sections were treated for 30 min in a mixture of 1 part methanol plus 1 part 30% H_2_O_2_ and 8 parts 80% Tris. Following pretreatment with 100% formic acid for 3 min to facilitate the immunoreactions, blocking with bovine serum albumin was performed to inhibit endogenous peroxidase and nonspecific binding. Subsequently, each of the sets was incubated for 18 h at 20 °C using the following commercially available primary antibodies: (**1**) a monoclonal anti-PHF-Tau antibody (1:2000; Clone AT8; Pierce Biotechnology, Rockford, IL, USA [Thermo Scientific]) for detection of abnormal tau protein in both pretangles and mature neurofibrillary changes of the Alzheimer-type. AT8 recognizes a phosphate-dependent epitope at Serine 202, Threonin 205, and possibly Serine 208 of the tau protein [57, 87] or (**2**) a monoclonal mouse antibody anti-beta-amyloid (1:5000; 1 mg/mL; Clone 4G8; Covance, Dedham, MA, USA) for Aβ. After processing with a corresponding secondary biotinylated antibody (anti-mouse or anti-rabbit IgG, 1:200; Linaris, Vector Laboratories, Burlingame, CA, USA) for 1.5 h, all immunoreactions were visualized with the avidin–biotin complex (ABC, Vectastain, Vector Laboratories, Burlingame, CA, USA) for 2 h and the chromogen 3,3’-diaminobenzidine tetrahydrochloride (DAB, D5637 Sigma, Taufkirchen, Germany). Omission of the primary antiserum resulted in non-staining. Positive as well as negative control sections were included.

The tissue sections were cleared and mounted in a synthetic resin with a refraction index of 1.58 (Histomount, National Diagnostics, Atlanta, GA, USA). Sections were viewed with a BX61 microscope (Olympus Optical, Tokyo, Japan), and digital micrographs were taken by H.B. using an Olympus XC50 camera together with the extended focal imaging (EFI) function (Cell D Imaging Software, Olympus, Münster, Germany). The EFI function was used for stacks of images taken at different optical planes. The algorithm extracts the image features with the sharpest contrast from all layers of the stack and merges them into a single image. Tau pathology and nerve cell loss were assessed semiquantitatively in all selected regions of all 308 cases by two observers (HB, MS) as follows: 0 (none/no detectable tau pathology), 0.5 (sparse or isolated changes), 1 (mild), 2 (moderate), 3 (severe changes). Neuronal loss in each region was determined based on the presence of extraneuronal lipofuscin granules and pigment-containing microglia as markers of lost pigment-laden nerve cells.

### Immunofluorescence (SYP + AT8, MAP2 + AT8) and confocal imaging

**Fig. 3:**
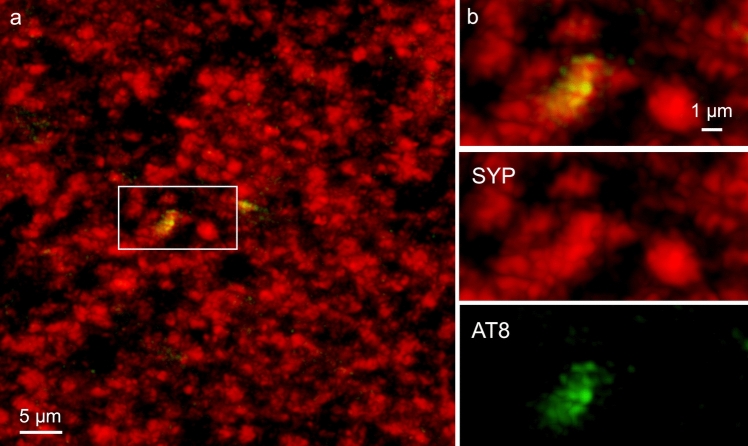
Immunofluorescence and AT8-positive dot-like structures. The first traces of abnormal tau in the cerebral cortex consistently occurred in anteromedial portions of the temporal lobe. **a**, **b** These consisted of a few AT8-immunopositive dot-like structures amidst many synaptophysin-positive terminal axonal boutons. **a** Molecular layer of the entorhinal cortex close to the transition to the transentorhinal cortex. Presynapses are immunolabeled against SYP1 (*red*). AT8 (*green*) marks abnormal tau. *Framed area* is shown at higher magnification in (**b**). **b** The AT8-immunopositive presynapse (dot-like structure is shown as a merge of the channels (upper one-third) and in the single channel representations (lower two-thirds). 100 µm section, male 73 years of age, NFT I, APOE unavailable

Fixed tissue from the hippocampal formation, including the entorhinal and transentorhinal regions, was sectioned at 100 µm on a vibratome (VT1200S vibratome, Leica, Wetzlar, Germany). The sections were then washed 1 × in PBS (Thermo Fisher Scientific, Waltham, MA, USA) and exposed to heat retrieval with 0.1 M citrate buffer (pH 6.0, 10 min, 100 °C). They then were incubated for 3 h in a blocking buffer at room temperature (3% BSA, Bio Froxx, Einhausen, Germany; 0.3% Triton X-100, Roche, Mannheim, Germany). Immunolabeling was performed for 1 d in a blocking buffer at 4 °C. The following antibodies were used: (**1**) a polyclonal chicken microtubule-associated protein MAP2A/B antibody (1:500, EnCor Biotechnology, Gainesville, FL, USA) as a dendritic marker; (**2**) a guinea pig polyclonal SYP antibody (1:500, Synaptic Systems, Göttingen, Germany) that recognizes presynaptic vesicles; (**3**) AT8 (1:500, see above). Subsequently, all sections were rinsed three times for 30 min 1 × in PBS. Incubation in blocking buffer with secondary antibody was performed with Alexa Fluor conjugated antibodies 488 (RRID:AB_2341099 for AT8), 647 (RRID:AB_2340379 for MAP2) and 647 (RRID:AB_2340476 for SYP) from Jackson ImmunoResearch Laboratories, Ely, UK, diluted 1:200 at room temperature for 2 h. The sections were subsequently rinsed again three times for 30 min. All steps from blocking onwards were performed under gentle agitation. The sections were mounted on slides with ProLong Gold antifade mountant (Invitrogen, Waltham, MA, USA). Confocal microscopy was performed by M.S. with Leica SP8 (for Fig. 3) or SPE (for Fig. 5) microscopes (Leica, Wetzler, Germany) as a three-dimensional image (finally shown as a projection image) with a 40 × objective (HC PL APO CS2 NA 1.30 for SP8 and ACS APO NA 1.50 for SPE, both oil immersion), pinhole aperture = 65.2 µm for SP8 and 98.5 µm for SPE. The images were then deconvolved using Huygens software (Scientific Volume Imaging, Hilversum, The Netherlands) and further processing of the images with adjustment of brightness and contrast in Imaris software (Oxford Instruments, Abingdon, UK).

### Statistical analyses

Descriptive statistics are presented as frequencies in the case of categorial variables; otherwise, the median and range are displayed. Chi-square, Mann–Whitney *U*, and Kruskall-Wallis tests were used to analyze between-group comparisons of categorical or ordered/metric variables. A two-sided, explorative type 1 error of 5% was assumed for all analyses. All analyses were conducted using the R software for statistical computing (version 4.3.2, www.r-project.org).

## Results

### Development of a plexus of abnormal tau-containing axon terminals mingled with similarly involved distal dendritic segments in the transentorhinal and entorhinal regions surrounding layer pre-α projection cells

**Fig. 4:**
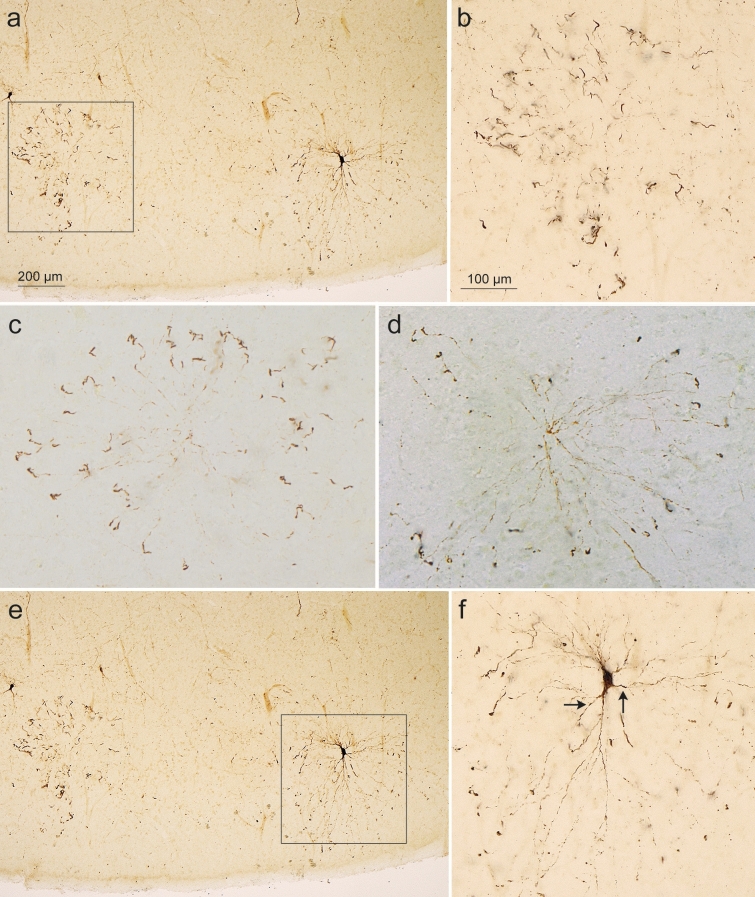
Involvement of layer pre-α in the entorhinal region in AT8 immunoreactions. **a**, **b** Shortly after the appearance of the dot-like particles, bent dendritic segments surrounding pre-α cells (lower left) in a skirt-like pattern were seen. Note swelling of the dendritic segments in (**b**)**.** Framed area in (**a**) is shown in greater detail in (**b**). Despite the absence of visible physical contacts between the center and the ‘skirt’ of AT8-immunoreactive dendrites at the perimeter, the impression emerges that they are not bundles of neuronal processes randomly belonging to multiple pyramidal cells. It can be assumed that the cell soma, to which the dendrites in (**c**) belong, is located in the center of the micrograph but is still free of abnormal tau. 100 µm section, male 45 years of age, NFT stage I, ε3/ε3. **c**, **d** In addition to the AT8-immunopositive dot-like structures, the distal dendritic processes filled with pretangle tau were among the first components in cortical projection cells to become involved. The distal dendritic processes increased in diameter and followed a curved course (**c**). Abnormal tau then extended into the proximal dendrites and marked a small portion of the soma (**d**). 100 µm sections, female 52 years of age, NFT I, ε3/ε4 (**c**) and male 77 years of age, NFT II, APOE unavailable (**d**).** e**, **f** Abnormal tau then marked the dendrites (arrow*s* in **f**) and the cell soma. Finally, pretangle tau also appeared in the axon (**f**) and could then be seen in the perforant path (see Fig. 7). *Framed area* in **e** is shown in greater detail in (**f**). 100 µm section, same individual as in (**a**, **b**). Scale bar in **a** applies to **c**, bar in **b** is valid for **c**, **d**, and **f**

Within the cerebral cortex, abnormal tau initially occurred in the transentorhinal or entorhinal regions only as AT8-positive dot-like (Fig. 3) and AT8-positive bent dendritic segments in the molecular layer and layer pre-α (Fig. 4). The AT8-immunopositive dendritic segments appeared to surround an individual (single) pyramidal cell (Fig. 4a–c) rather than to originate from multiple overlapping pyramidal cells. A skirt-like arrangement of bent distal segments of the dendrites permitted recognition of a central AT8-immunonegative area, around which the skirt of immunopositive distal dendritic segments was radially oriented.

The AT8-positive distalmost dendrites displayed local varicose enlargements and constrictions [11] (Fig. 4a, b). Once concomitant alterations were present in nearly all of the distal dendrites generated from a single pre-α cell (Fig. 4c), traces of abnormal tau in the proximal dendrites were seen (Fig. 4d) that then filled the cell soma and, finally, even the axon (Fig. 4e, f). These abnormalities in individual nerve cells developed near closely adjacent cells of the same type that were devoid of abnormal tau.

**Fig. 5:**
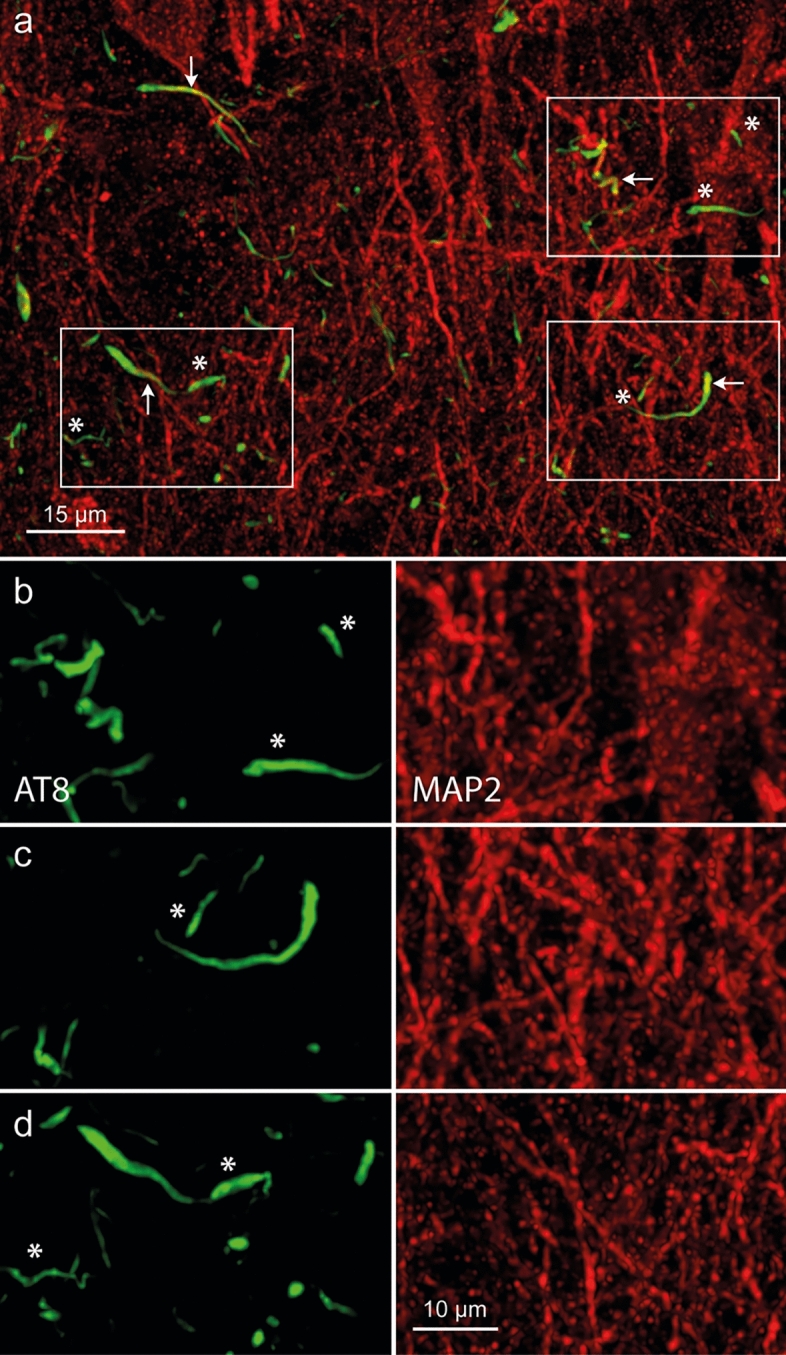
Immunofluorescence and AT8-immunopositive ‘ghost threads’.** a** Overview of the molecular layer in the entorhinal region. Neuronal cell somata and dendrites are immunolabeled in *red* (MAP2). AT8 (*green*) marks abnormal tau. Framed areas are shown at higher magnification in (**b**–**d**). *White arrows* indicate MAP2-immunopositive neurites shown as a merge of the two immunolables in **a** and in the single channels (**b**–**d**), whereas *white asterisks* mark MAP2-immunonegative ‘ghost threads’ in the neuropil (no overlap with the MAP2 signal in the merge). Ghost threads have not been described previously. 100 µm section, female 73 years of age, NFT III, ε2/ε4

**Fig. 6:**
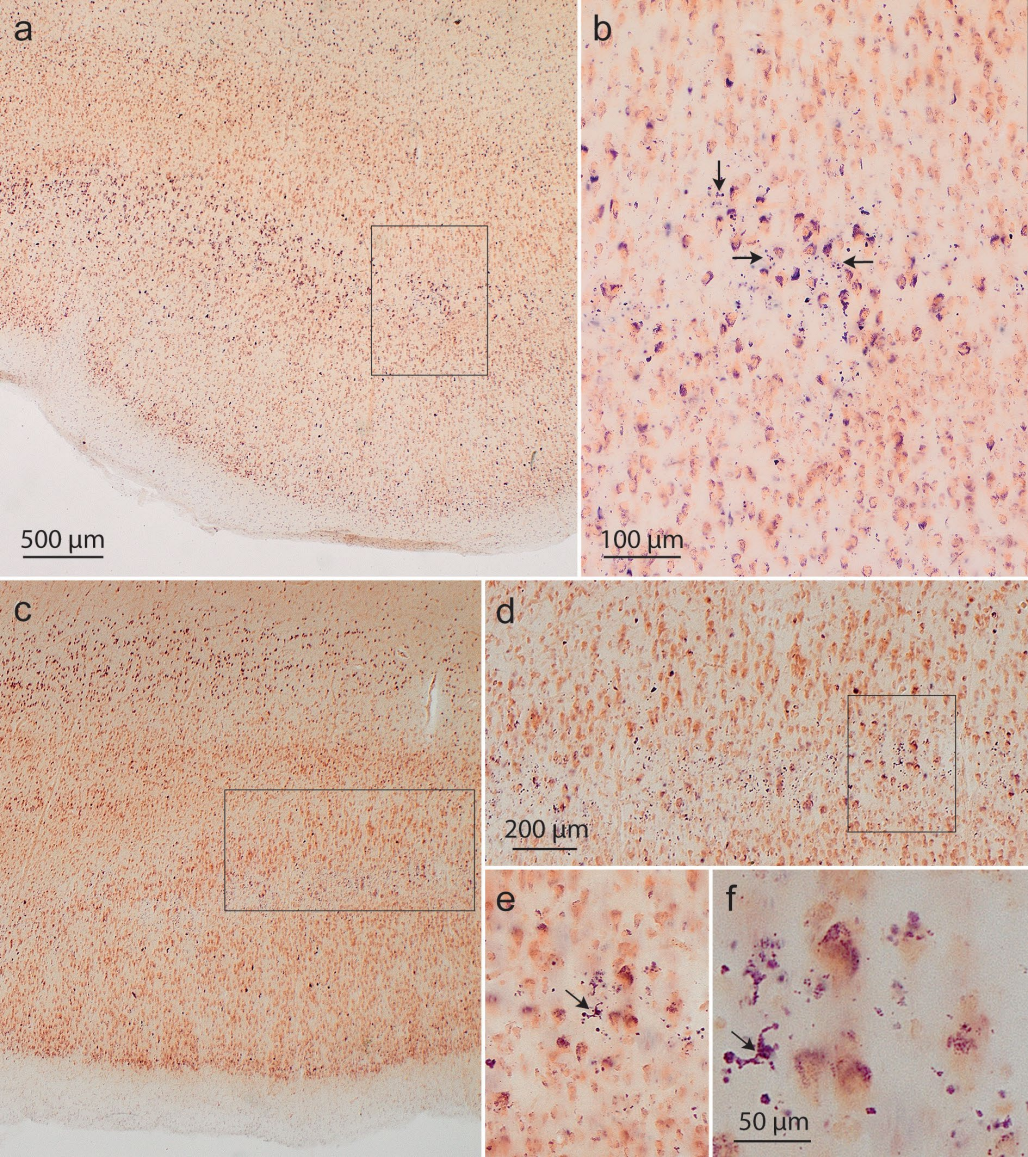
Tau-related loss of nerve cells in staining with aldehyde fuchsine and Darrow red. AD-related loss of nerve cells as indicated by aldehyde fuchsine-positive lipofuscin granules from dead pigment-laden neurons cells. **a** Overview of the deep terminal portion of layer pre-α in the lateral transentorhinal region showing the strongly pigmented pyramidal cells of this layer at mid-cortical level. Framed area is shown in greater detail in **b**. **b** This layer shows the first signs of AD-specific nerve cell loss, here indicated by extraneuronal accumulations of neuronal lipofuscin remnants in the neuropil (arrows). 100 µm section, male 72 years of age, NFT stage III, ε3/ε4. **c** Overview of the transentorhinal region. *Framed area* is displayed at higher magnification in **d**, and framed area in **d** at higher magnification in **e**. **e**, **f** Lipofuscin granules can be taken up by microglial cells. *Arrows* point to a microglial cell containing lipofuscin granules from nearby dead pyramidal cell(s). 100 µm section, male 68 years of age, NFT III, APOE unavailable. Scale bar in **a** applies to **c**, bar in **b** to **e**

The pretangle tau in dendrites gradually became fibrillar neuropil threads (NTs). After the death of the host neuron, these dendritic processes lost their ensheathing cellular membrane and persisted, in analogy to ghost tangles, as ‘ghost threads’ (Fig. 5). The tau inclusions in the cell somata converted into flame- or star-shaped NFTs (Suppl. Fig. 2). Tangle-bearing nerve cells can survive for a long time but ultimately die [93]. The NFTs then developed into ghost tangles, and the neuronal lipofuscin granules from the dead cells lay exposed temporarily in the extracellular space (Fig. 6a, b), where they were phagocytosed by microglia (Fig. 6c–f). The gradual progress of neuronal loss (based on the presence of extraneuronal lipofuscin granules and pigment-containing microglia as markers of lost pigment-laden nerve cells) could be similarly followed in other regions affected during the AD-related pathological process (Suppl. Fig. 3).

**Fig. 7:**
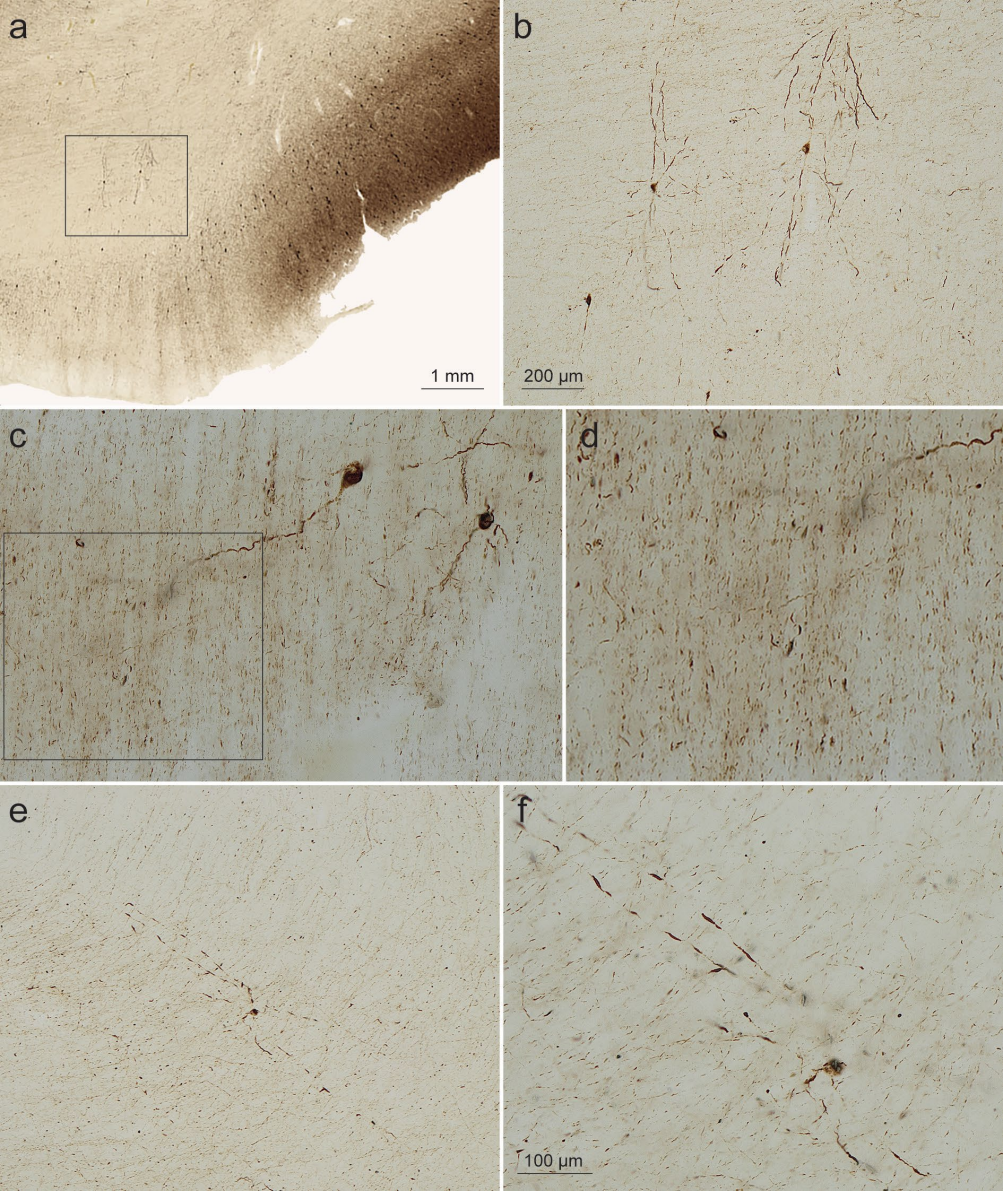
Abnormal tau in perforant path axons and interneurons in AT8 immunoreactions. **a**, **b** The perforant path in the white matter subjacent to the subiculum. *Framed areas* in **a**, **c** (axons) are shown in greater detail in **b**, **d**. At this location, a few AT8-positive interneurons regularly occurred (two are shown in **b** and **c**, one in **e** and** f**) and were characterized by very long, unbranched and smoothly contoured dendrites; **e** is shown in **f** at higher magnification. The dendrites were oriented at right angles to the course of the AT8-positive axons. 100 µm sections, male 78 years of age, NFT stage III, ε3/ε4 (**a**, **b**); female 73 years of age, NFT stage III, APOE unavailable; (**c**, **d**); male 71 years of age, NFT stage III, ε3/ε4 (**e**, **f**). AT8 immunoreactions. Scale bar in **b** is valid for **e**, bar in **f** also applies to **c**

Involved axons of the projection cells in the outer entorhinal layers forming the perforant path beneath the subiculum and heading for the hippocampal formation ran parallel to each other, these axons characteristically contained pretangle tau for short distances, interrupted by longer gaps lacking abnormal tau [123] (Fig. 7a, b). Between bundles of abnormal axons of the perforant path lay disseminated a few pretangle-containing interneurons with very long, smoothly contoured dendrites oriented at nearly 90 degrees to the fibers of the perforant path in the white matter subjacent to the subiculum (Fig. 7c, d). These interneurons were consistently found at this location (Fig. 7e, f).

### Development of a plexus of abnormal terminal axons and distal dendrites surrounding pyramidal cells of the prosubiculum, bifurcating into both the stratum oriens and stratum lacunosum of CA1 and CA2

**Fig. 8:**
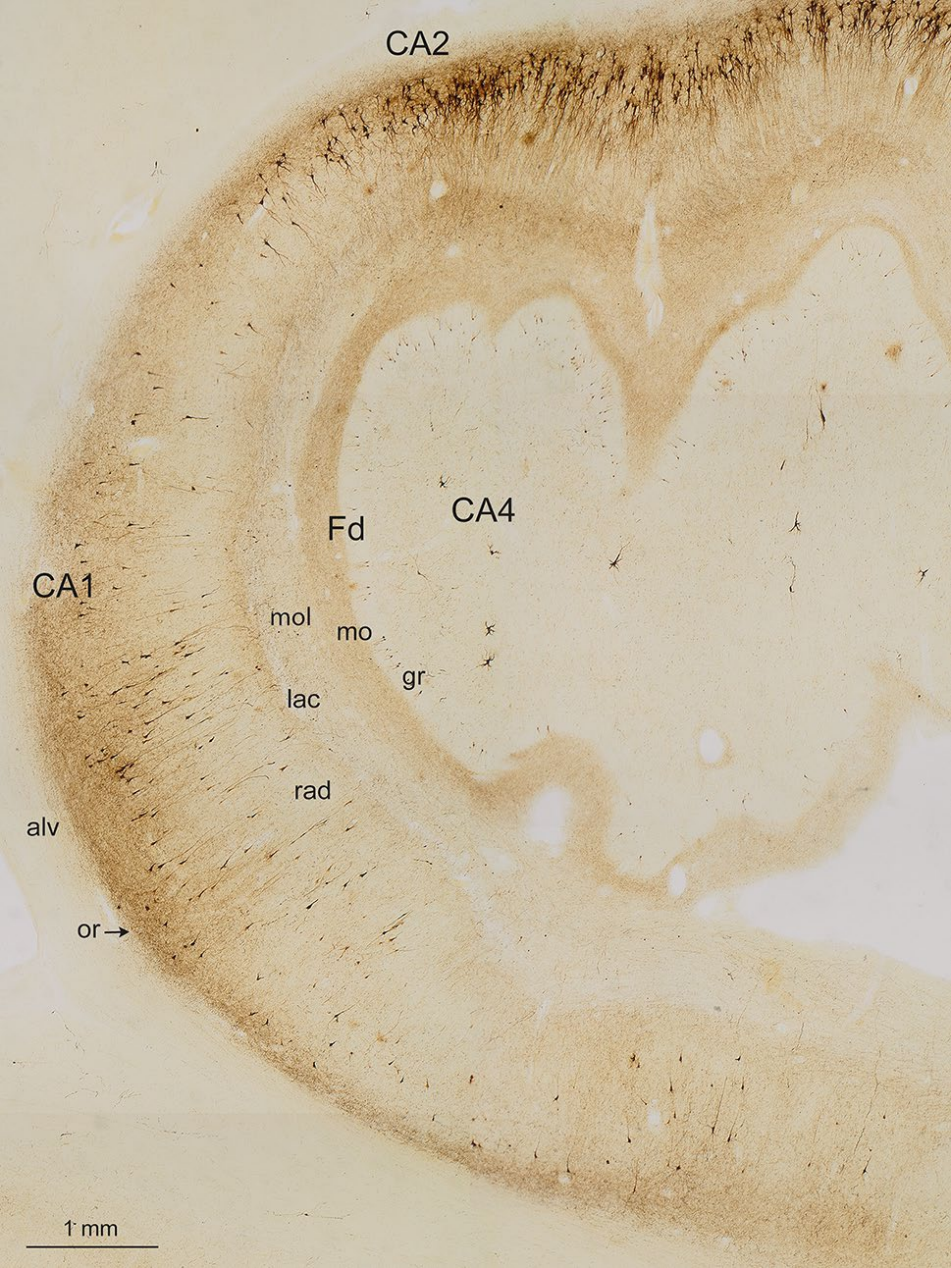
Overview of the human Ammon’s horn (CA1-CA4) and the dentate fascia (Fd) in an AT8-immunoreaction. The figure displays the two stripes of accumulated affected nerve cells processes in the stratum oriens and stratum lacunosum of CA1 (or, lac). In CA2, the stripes in the stratum oriens and stratum lacunosum approximate each other. There is neither a stratum lucidum nor a clearly recognizable stratum radiatum in CA2. The pathological process has sufficiently progressed to show the essential formations of early AD-related lesions, including not only the affected projection neurons filled with pretangle tau and/or NFTs/NTs but also the diseased axon terminal boutons and affected distal dendritic segments in the stratum oriens and stratum lacunosum of CA1 and CA2 as well as in the outer two-thirds of the dentate molecular layer. The pyramidal layer of CA1 shows numerous AT8-positive pyramidal cells. The myelin-rich alveus and the stratum radiatum display only sparse immunoreactions (al, rad). The broad molecular layer between stratum lacunosum and the obliterated hippocampal fissure (mol) similarly is poor in AT8-positive structures except for the pathological enlargements of the terminal apical dendrite of CA1 pyramidal cells. In addition, the terminal zone of the perforant path in the middle and outer two-thirds of the dentate molecular layer is marked by an abundance of immunopositive axons (mo). The granular layer and the inner third of the molecular layer of the dentate fascia are only sparsely affected (gr). Involved cells in CA2 are more densely packed than in CA1 and, here, formed a CA2 focus. The CA4 sector shows only a few affected multipolar mossy cells. 100 µm, AT8 immunoreaction, male 72 years of age, NFT stage III, ε3/ε4

AT8-positive axons of the perforant path accumulated in the prosubiculum and, from there, axon collaterals bifurcated into two stripe-like plexus, one within the CA1 and CA2 stratum oriens, the other, less dense, in the CA1 and CA2 stratum lacunosum without wide extensions into their molecular layer (Tables 3 and 4, Fig. 8). The conspicuous AT8-positive plexus within the stratum oriens displayed a relatively sharp border to the alveus but gradually decreased in density towards the pyramidal layer. At the border between CA1 and CA2, the two stripes once again approximated each other and formed, together with the pyramidal layer of CA2, a broad zone of AT8-positive cellular processes (Fig. 8).

**Fig. 9:**
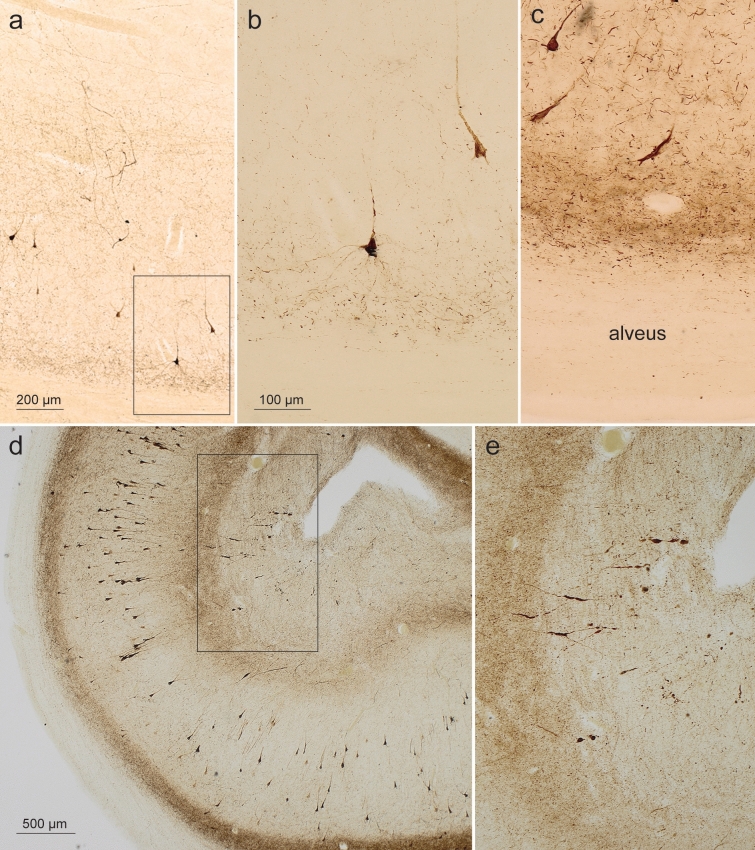
AT8-positive pyramidal cells (CA1, stratum oriens) and AT8-positive spindles in the stratum moleculare of CA1. **a**, **b** Pretangles developed in basal dendrites (stratum oriens) and side branches (stratum lacunosum) of the apical dendrite of CA1 pyramidal cells. They then filled the somatodendritic domain of terminal segments of the basal dendrites of CA1 pyramidal cells. *Framed are* in **a** is shown in greater detail in (**b**) 100 µm section, male 57 years of age, NFT stage II, ε3/ε3. **c** Finally, axons of the affected cells were filled and appeared as short, thin lines in the myelin-rich alveus (and, ultimately, the fornix; not shown). 100 µm section, male 69 years of age, NFT stage III, APOE unavailable. **d**, **e** The distal apical dendrite of CA1 pyramidal cells developed AT8-positive spindle-shaped varicosities during the early disease phase. *Framed area* in **d** appears at higher magnification in **e**. 100 µm section, male 78 years of age, NFT III, ε3/ε4. *Scale bar* in **b** is valid for **c** and **e**

As in the transentorhinal/entorhinal regions, the first traces of abnormal tau in the Ammon’s horn were seen in the CA1 stratum oriens in the form of dot-like AT8-positive axonal terminal boutons (not shown). We postulated that the dot-like structures represent collateral branches of the perforant path that contribute to the formation of the stratum oriens. These changes in CA1 were followed by the development of pretangles in all distal segments of both basal dendrites (stratum oriens) and side-branches (stratum lacunosum) of the apical dendrite of pyramidal cells (Fig. 9a, b) that formed the two stripe-like plexuses (Fig. 8). Proximal dendrites and then the cell somata of involved cells became filled with abnormal tau that developed into fibrillar NTs/NFTs (Fig. 9b). Finally, the axons of the involved CA1 cells became AT8-positive and appeared as part of the myelin-rich alveus and, lastly, the fornix (Fig. 9c and see also Fig. 8).

A unique feature of affected CA1 pyramidal cells during AD is that some developed AT8-positive spindle-shaped varicosities and constrictions within their distalmost apical dendrites in the stratum moleculare [10] (Fig. 9d, e). These deformed segments (a sizeable portion of the apical dendrite) finally lost their connection to the host cell soma.

**Fig. 10:**
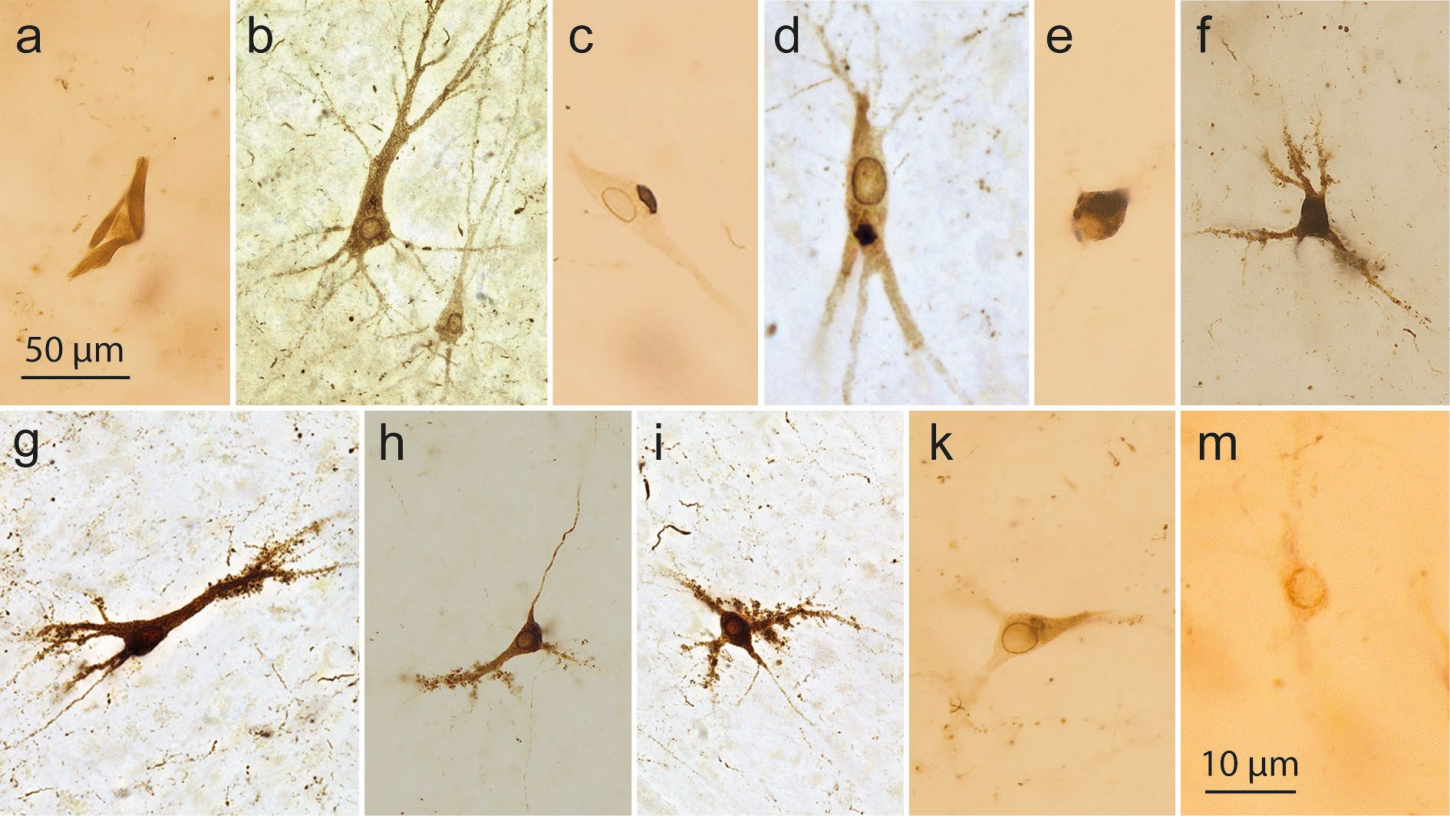
AT8 immunoreactivity in Cornu Ammonis cells. **a CA1** ghost tangle (an extraneuronal NFT) marks the location of a lost pyramidal cell, male 93 years of age, NFT III, ε3/ε3. **b–e CA2 pyramidal cells.** CA2 cell (at left) with an AT8-positive perinuclear rim, female 93 years of age, NFT III, ε3/ε3. **c** CA2 cell with perinuclear rim plus aggregated tau ‘packet’, female 69 years of age, NFT II, APOE unavailable. **d** CA2 cell with perinuclear rim and tau ‘packet’, female 77 years of age, NFT II, ε3/ε4. **e** CA2 cell with a globose tangle, male 92 years of age, NFT III, APOE unavailable. Globose tangles occur in CA2-CA4 and in the granular cells of the Fd, but not in CA1. The perinuclear rim possibly influenced the consolidation of pretangle tau into a globose tangle close to the cell nucleus. **f**–**k** CA3/CA4 multipolar mossy cells. **f** CA4 excrescence-bearing mossy cell, female 52 years of age, NFT I, ε3/ε4. **g** CA3 mossy cell with excrescences, male 75 years of age, NFT III, ε2/ε4. **h** CA4 mossy cell with excrescences and axonal tau, male 62 years of age, NFT I, ε3/ε3. **i** CA4 mossy cell with perinuclear rim plus excrescences, male 75 years of age, NFT III, ε2/ε4e. Same individual as in **g**. **k** CA4 mossy cell with perinuclear rim and tau ‘packet’, male 93 years of age, NFT III, APOE ε3/ε3. Same individual as in **a**. **m** Fd cell with an AT8-positive perinuclear rim, male 57 years of age, NFT III, ε3/ε3. 100 µm sections. AT8 immunoreactions. The micrographs for **b**, **d**, **g**, and **i** were taken by M.S. using a Keyence BZ-X800 series microscope (Keyence Deutschland Inc., Neu-Isenburg, Germany) to acquire z-stack images together with the Keyence Analyzer software Full Focus. The Full Focus function was used to remove out-of-focus image points and generate a projection image of all focus points. Scale bar in **a** is valid for **b**–**k**

As reported elsewhere [25, 86, see also 55, 60, 71], the development of pretangle tau in CA2 pyramidal cells differed from that in CA1: A thin but marked perinuclear rim of abnormal tau developed in CA2 pyramidal cells, a feature that helps to distinguish between pyramidal cells of CA1 and CA2 (Fig. 10b–d). The soluble pretangles were uniformly distributed throughout the somatodendritic domain; however, in CA2 some pyramidal cells also displayed compressed ‘packets’ of abnormal tau with rounded contours (Fig. 10c, d).

### Occurrence of pretangle tau in thorny excrescences of CA3 and CA4 projection cells

The CA3 sector contained reduced numbers of abnormal tau-containing neuronal processes in the stripe-like zones below and above the pyramidal layer. The stripes were replaced by pretangles in the thorny excrescences (spines) along the proximal dendrites of CA3 and CA4 mossy cells [9] (Fig. 10f–i). All excrescences of one and the same nerve cells appeared to become AT8-positive simultaneously (Fig. 10f–i). Assemblies of involved excrescences were also observable in isolation [25].

The pretangle tau filled the dendrites and gradually tapered off distally, again followed by changes in the cell somata and finally the axon. The occurrence of involved pyramidal cells in the prosubiculum and CA1/CA2 sectors consistently preceded the occurrence of tau inclusions in the CA3/CA4 sectors (Tables 3 and 4). Again, a perinuclear rim of pretangle tau was present in CA3/CA4 mossy cells [86] (Fig. 10h–k). With time, the soluble pretangles were no longer visible in the dendrites and probably converted to fibrillar tau nearby the cell nucleus to form a globose tangle. We did not see ghost tangles in CA3/CA4 sectors during NFT stages I–III. Sector CA4 contained, in addition to the predominating mossy cells, a few AT8-positive and lipofuscin-rich cells (interneurons) that had lengthy and smoothly contoured dendrites. These cells displayed neither AT8-immunoreactive perinuclear rims nor thorny excrescences.

**Table 3:**
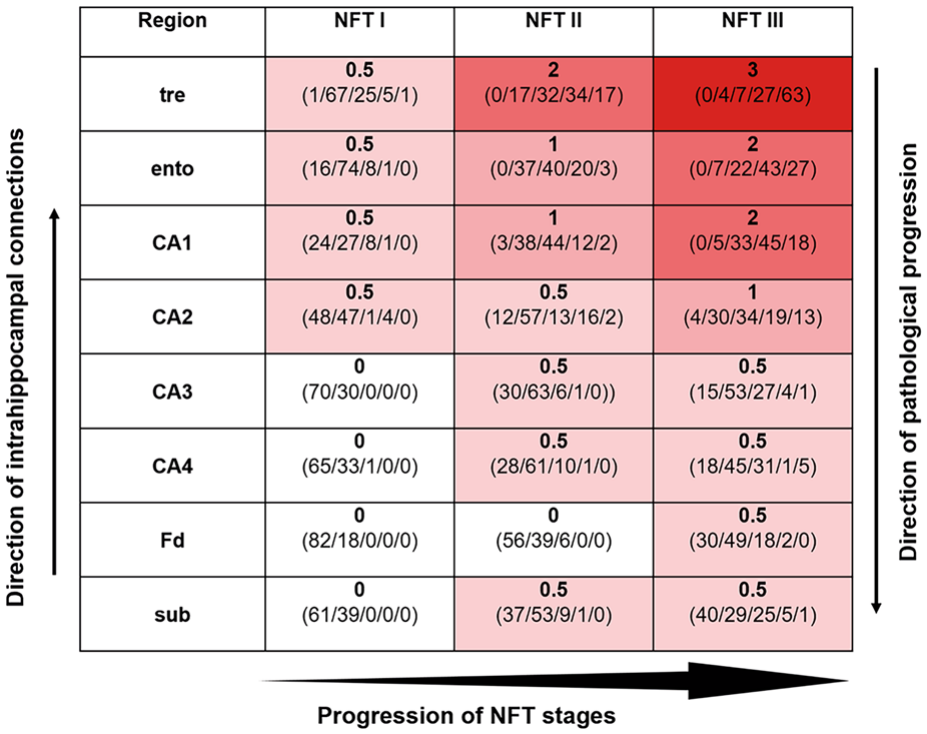
Eight involved anatomical regions in cases with early (NFT stages I–III) tau changes

The density of all AT8-positive lesions (pretangle tau, NFTs, NTs) taken together was semiquantitatively assessed as 0 = none, 0.5 = subtle, 1.0 = mild, 2.0 = marked, and 3.0 = severe. Statistics are given as median (bold type, 0 = *white*, 0.5 = *pink*, 1 = *dark pink*, 2 = *red*, or 3 = *dark red*) and percentage of cases with single tau ratings ranging from 0 to 3 (in parentheses, single ratings separated by/and adding up to 100% in each cell). Each Kruskal–Wallis test for the eight regions shown (always comparing NFT I vs. NFT II vs. NFT III) achieved a significant *p*-value of < 0.001). *Black arrow* at the left shows the known connectivities within the hippocampal formation; the *black arrow* at right indicates the sequence of pathological tau progression proposed in the present study

*tre* transentorhinal region, *ento* entorhinal region, *CA1-CA4* cornu Ammonis (Ammon’s horn) sectors 1–4, *Fd* dentate fascia, *sub* subiculum

**Table 4:**
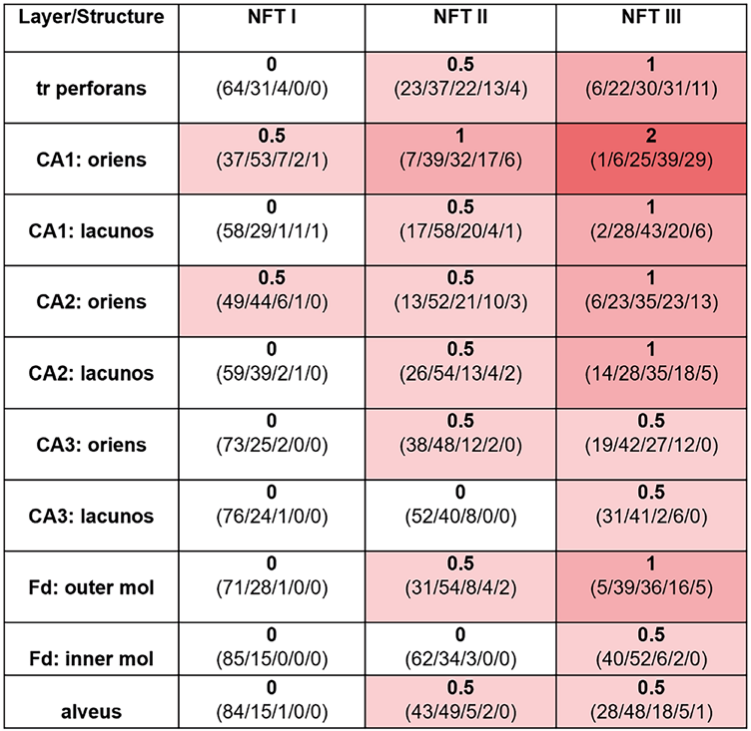
Layers/Structures affected in cases with early tau morphological changes according to NFT stages

The percentage of cases showing the presence of AT8-positive lesions in specific structures is given according to each NFT stage. The density of all AT8-positive inclusions taken together was semiquantitatively assesed as 0 = none, 0.5 = subtle, 1.0 = mild, 2.0 = marked, and 3.0 = severe. Statistics are given as median (bold type, 0 = *white*, 0.5 = *pink*, 1 =* dark pink*, 2 = *red*, or 3 = *dark red*; here not applicable) and percentage of patients with single tau ratings ranging from 0 to 3 (in parentheses, single ratings separated by/and adding up to 100% in each cell). The local structures with abnormal morphological changes displayed in relationship to each structure a significant difference (Kruskall-Wallis test *p*-value of < 0.001) between NFT stages I, II, and III

*tr perforans* perforant path (tractus perforans), *CA1-CA3* cornu Ammonis (Ammon’s horn) sectors 1–3, *oriens* stratum oriens, *lacunos* stratum lacunosum, *Fd: outer mol* dentate fascia, outer molecular layer, *Fd: inner mol* dentate fascia, inner molecular layer, *alveus* alveus hippocampus (white matter)

### Development of abnormal tau in granular cells of the dentate fascia

With the development of pretangle tau in perforant path axons, a plexus of dot-like AT8-positive terminal endings developed in the middle and outer two-thirds of the dentate molecular layer [25, 121, 127] (Fig. 8 mo). Subsequently, a few dot-like structures also developed within the inner one-third of the molecular layer. In parallel with this late change, slender dentate granular cells developed a perinuclear rim of pretangle tau (Fig. 10m). Abnormal tau was also found evenly disseminated throughout the somatodendritic domain. Granular cells with abnormal tau were not altered in shape.

### Late-developing abnormal changes

**Fig. 11:**
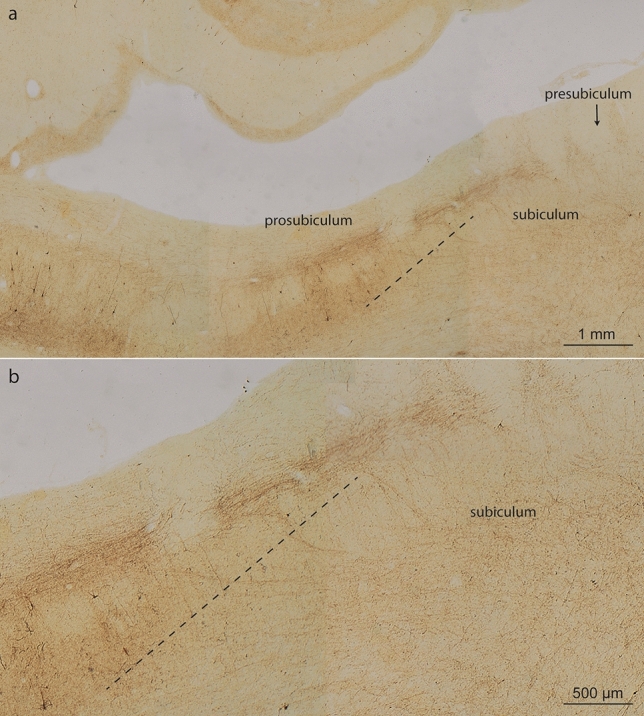
Views from the CA1 sector of the prosubiculum, subiculum and presubiculum. **a** The wedge-shaped prosubiculum is located between the CA1 sector and the subiculum. The hatched black line is intended to aid in distinguishing the prosubiculum from the subiculum. The subiculum appears on cross-sections as a wing-shaped structure and is dominated by large pyramidal cells furnishing output pathways from the Ammon’s horn. **b** In NFT stage III, the region remains almost intact or displays only a few pyramidal cells with pretangles. Here, only AT8-positive axons are seen. Small bundles of AT8-positive axons belonging to the perforant path penetrate the subiculum and run into the prosubiculum, where they split into two stripes: a superficial and relatively thick stripe that heads to the stratum lacunosum of CA1, and a deep stripe that maintains contact to the subicular surface and heads towards the CA1 stratum oriens (above the *hatched black line*). In pigment-Nissl sections, the subicular boundaries are reliably recognized because cells of the outer subicular pyramidal layer are known to display unusual accumulations of lipofuscin granules within the middle portions of their apical dendrite [23]. 100 µm, AT8 immunoreaction, same individual as in Fig. 8

In the subiculum, abnormal tau was seen late (Fig. 11). Subicular involvement peaked as the latest feature of the AD-related hippocampal process [23] and was accompanied by a gradual increase of AT8-positive axons in both the alveus and the fornix (Tables 3, 4, Figs. 8 alv and 11).

In sum, the associations between each of the NFT stages I, II, and III, in the absence of Aβ deposits, and both the eight involved anatomical regions as well as the local layers and structures within each of the eight regions studied were significant (*p* < 0.001) (Tables 3 and 4).

### Tau-related loss of involved nerve cells during NFT stages I–III

An insidious onset of nerve cell loss accompanied NFT stages I–III, as indicated by the presence of extraneuronal NTs/NFTs in astrocytes (not shown) and neuronal lipofuscin granules in microglial cells (Fig. 6f). Initially, pigment-laden microglial cells as a sign of nerve cell loss were hardly recognizable but became more distinct as the pathological process progressed (Table 5). The Kruskal–Wallis test showed significant regional differences (*p* < 0.001 for the regions tre/ento, CA1 and CA2, and *p* = 0.003 for CA3/CA4/Fd) with regard to neuronal loss in NFT stages I–III (Table 5).

Neuronal loss followed the same sequence as that seen in the development of the tau changes. Thus, the first signs of nerve cell loss occurred in the lateral transentorhinal area (Fig. 6a), directly abutting the temporal neocortex [13]. The densely pigmented pre-α cells at this location contrasted sharply with the accompanying but markedly less strongly pigmented neocortical pyramidal cells. Lost nerve cells at this site were indicated by pigment-laden microglia (Fig. 6f).

**Fig. 12:**
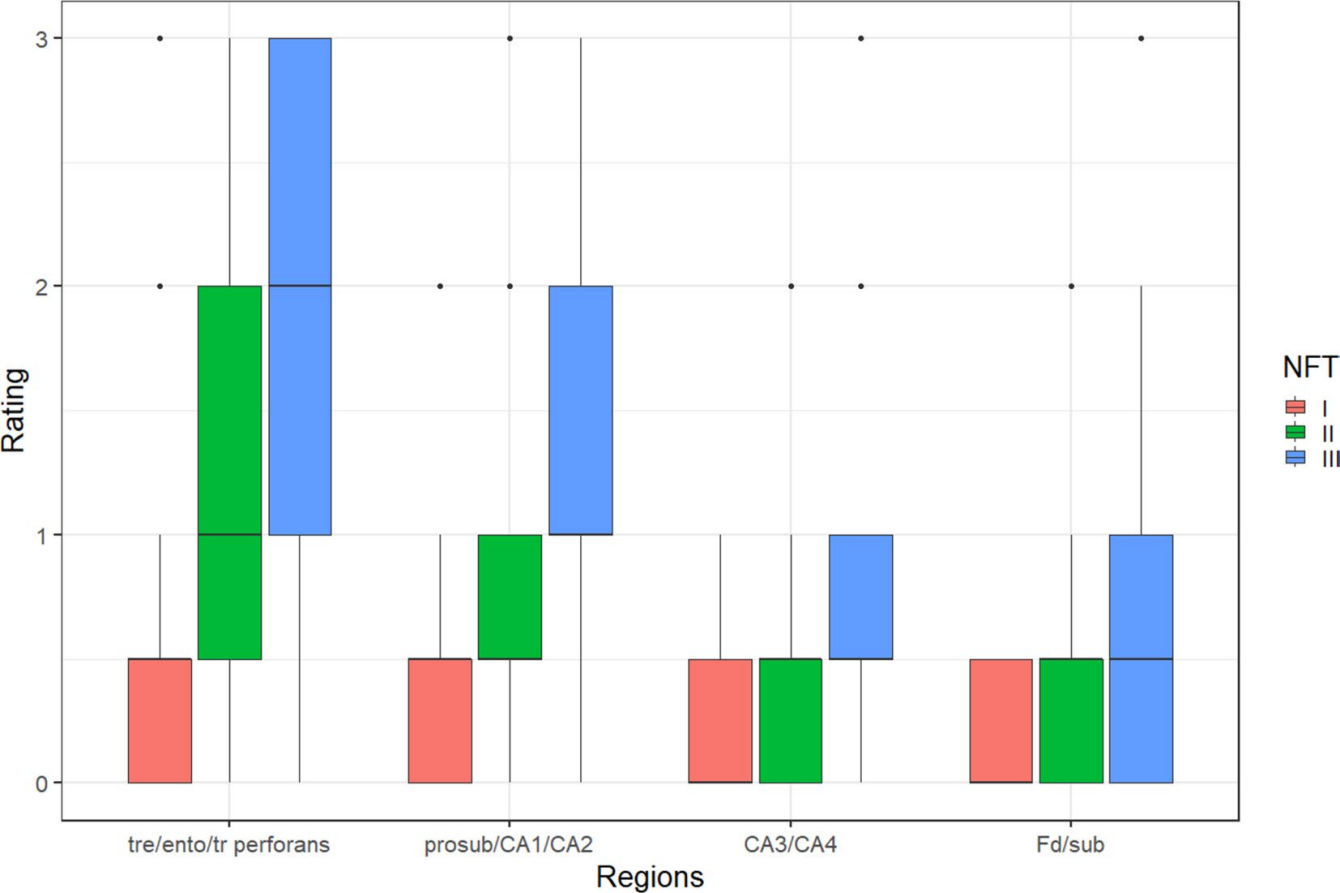
Diagram showing regional trajectory of tau morphological changes by NFT stage from (1) the tre/ento/tr perforans via the perforant path to (2) procubiculum/CA1/CA2, to (3) CA3/CA4, and to (4) the Fd and subiculum. The density of all AT8-positive lesions (pretangle tau, NFTs, NTs) in each of the four groups taken together was semiquantitatively rated as 0 = none, 0.5 = subtle, 1.0 = mild, 2.0 = marked, and 3.0 = severe to evaluate the possible regional trajectory of tau progression. The Kruskal–Wallis test comparing the four groups reached significance (*p* < 0.001)

The ordered progression of morphological changes at each location according to four groups also suggested a potential sequence of focused point-to-point transneuronal tau spreading, i.e., anterogradely from (**1**) involved entorhinal pre-α cells to (2) pyramidal cells of the prosubiculum, CA1, CA2, from there to (3) mossy cells in CA3 and CA4 and, finally, from mossy cells to (4) dentate granular cells and subiculum. The Kruskall-Wallis test results with regard to a possible trajectory of transneuronal tau spreading reached significance (*p* < 0.001) (Fig. 12).

## Discussion

The present study is based on the varying regional extent of the AD-related AT8-positive pathology with different degrees of severity that evolves in specific regions of the temporal allocortex The progressive tau changes develop in a stereotypic and systematic manner [23, 42, 65, 66, 86, 92, 95, 124]. The average age of the cases increased from 57.4 years in NFT stage I to 69.9 years in stage II, and 75.3 years in stage III (Table 1, Fig. 2a–c) in concert with the progression of the tau changes.

The earliest changes in stage I cases consist of barely a few AT8-positive dots in isolated axon-like structures and of pretangles in abnormally bent dendritic segments in the transentorhinal/entorhinal layer pre-α [13, 20, 25] (Figs. 3, 4). Given their size, the AT8-positive dots possibly were pathologically changed terminal axonal boutons generated by affected subcortical nuclei projecting to this cortical field, whereas the abnormally bent dendritic segments could represent axonal terminals combined with abnormally changed AT8-positive distalmost dendritic segments of pre-α projection neurons [13, 20, 115] Only a few subcortical nuclei are known to become involved prior to AD-related cortical changes and these include the locus coeruleus, the upper raphe nuclei, and magnocellular nuclei of the basal forebrain [8, 21, 23, 46, 62, 74, 100, 111]. The projections of these subcortical nuclei to portions of the cerebral cortex are usually described as ‘diffuse’ or ‘widely dispersed’ [8, 23]. Our findings, however, suggest a more highly focused projection from defined regions of the nuclei to circumscribed portions of the cortex.

Currently, the transmission of the pathological process from one nerve cell to another is thought to proceed via axonal seeds of abnormal tau anterogradely transferring the disease to dendrites of neurons following in the neuronal chain [1, 31, 37, 53, 58, 59, 77, 81, 82, 94, 105]. The entorhinal pre-α neurons initially display abnormal tau in only the distal segments of their dendrites (Fig. 4b). Inasmuch as no other portions of an affected cell contain abnormal tau, we and others have concluded that pretangle tau originates from localized sources of tau in dendrites [24, 72, 73, 91, 113, 114]. The tau inclusions cause alterations in the shapes of the distal dendrites and possibly stimulate a renewed growth that could explain their final size and tortuosity [11]. Thereafter, the proximal dendrites, the cell soma, and the axon become filled with soluble pretangle tau (Fig. 4f), which may be toxic [78]. With time, the visibility of the dendrites becomes reduced, perhaps because the soluble pretangle tau that generally is uniformly dispersed in the cytoplasm gradually aggregates, consolidating into insoluble and less toxic NTs and NFTs. It remains an open question whether at the same time a reduction in the length and number of dendritic processes occurs.

The AT8-positive axons of pre-α cells that contribute to the formation of the perforant path do not display severe axonal abnormalities (Fig. 7d), nor does the presence of abnormal tau appear to severely damage their axonal cytoskeleton. The abnormal tau within the axon, therefore, is unlikely generated from the axonal cytoskeleton itself [71]; rather, it represents soluble abnormal tau that has been transferred into the axon from the cell body via the axon hillock [24, 25].

The presence of AT8-positive interneurons interspersed throughout the perforant path has not been described earlier (Fig. 7). Although nothing is known about their functions, they appear to be ‘strategically’ positioned. In general, the AD-related pathological process preferably targets projection neurons, by and large sparing local circuit neurons or interneurons with a short axon [23]. It is unknown why this special type of perforant path interneuron consistently develops abnormal tau. It is possible that the initially soluble pretangle tau within the involved interneurons is strongly toxic [78, 98]. This could explain why these cells are incapable of developing the less soluble and less toxic neurofibrillary lesions. Instead, they perish shortly after the appearance of abnormal intraneuronal tau. In analogy to ghost tangles, ‘ghost interneurons’, are unknown.

**Fig. 13:**
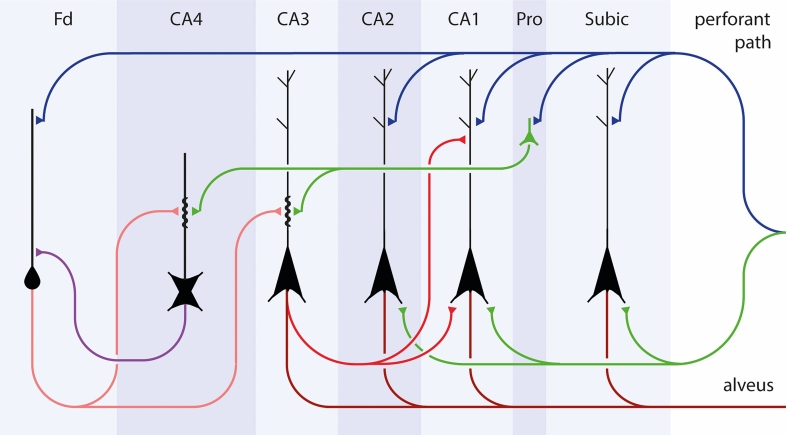
Summary diagram of the here-proposed disease-related intrahippocampal sequence of tau progression. We interpret our findings to mean that a sequence of neuronal involvement in AD could proceed from the entorhinal region to the hippocampal prosubiculum and CA1/CA2 sectors (*blue and green connections*), then to the thorny excrescences of mossy cells in CA3/CA4 (*green connections*), whence it reaches the dentate granular cells (*violet*) [25]. The hypothetical pathways appear in *green*

It has been reported that an unidirectional sequence of connections leads from dentate granule cells to CA1 within the hippocampal formation [70, 122, 127] (Table 3, *black arrow at left*). Nonetheless, the axons of these known pathways (stratum lucidum mossy fibers, stratum radiatum Schaffer collaterals) remain virtually devoid of AD-related pathology or display only subtle changes [23]. Previously, we hypothesized a sequence of neuronal involvement in AD that proceeds, in part, in the opposite direction [25] (Table 3, *black arrow at right*). According to our hypothesis, the disease-related intrahippocampal sequence starts within collaterals of the perforant path that connect the entorhinal region with the hippocampal prosubiculum and CA1/CA2 sectors (Fig. 13, *blue and hypothetical green connections* to the stratum oriens). Thereafter, we speculate that it reaches the thorny excrescences and mossy cells in CA3/CA4 (Fig. 13, *green connections*, still unverified in primate studies) and, subsequently, via known connections the dentate granule cells (Fig. 13, *violet connection at left*). Although the green connections proposed here are still uncertain, nonetheless, the statistical results for the here-proposed directionality of tau progression within the hippocampal formation were significant (*p* < 0.001) (Fig. 12).

**Fig. 14:**
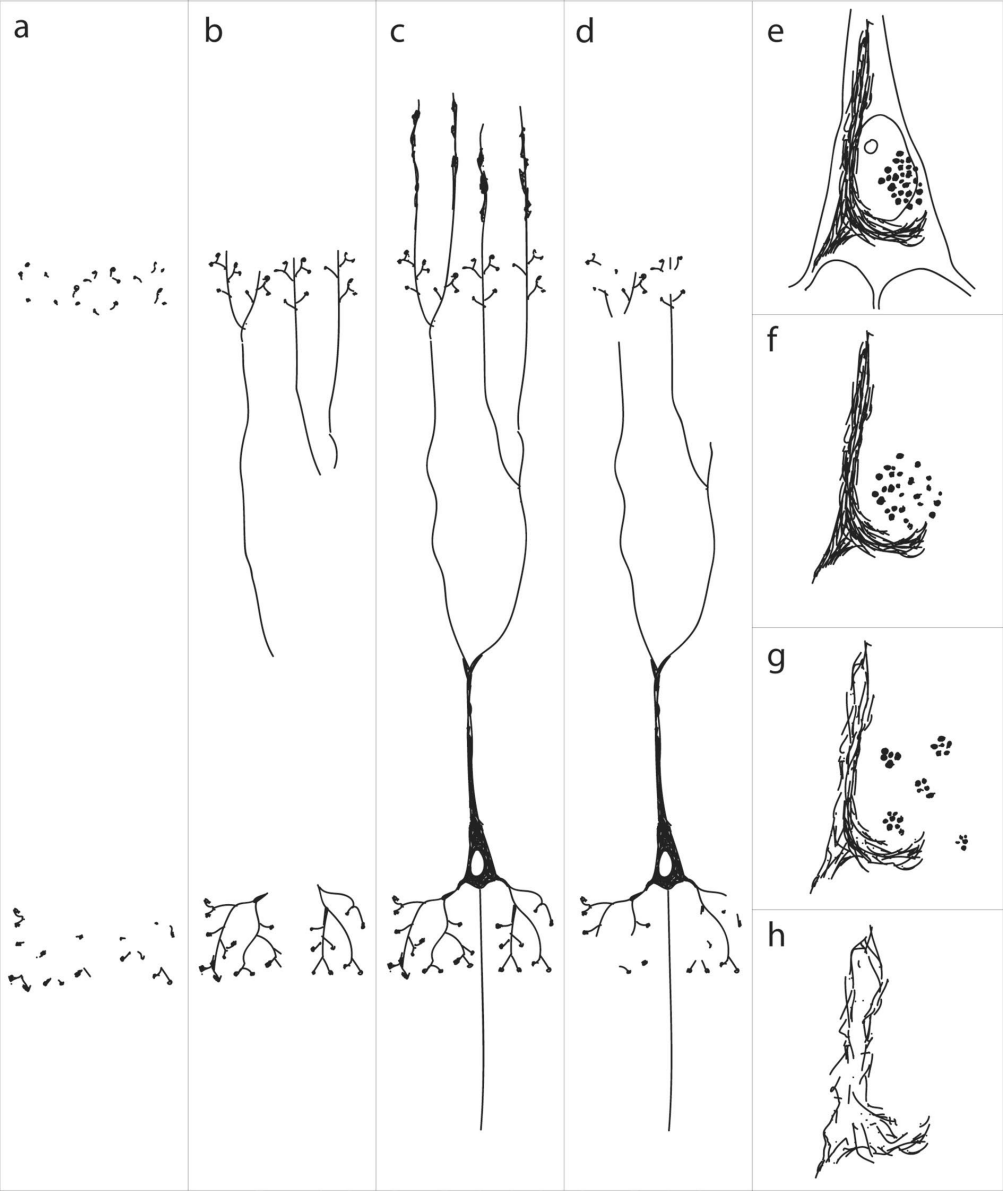
Sketches of the here-proposed sequential development of abnormal tau in CA1 pyramidal cells.** a** Initially, abnormally bent distalmost dendritic segments of involved pyramidal cells become filled with AT8-immunoreactive tau aggregates. **b** Next, pretangle tau emanates from the distal segments into the proximal dendrites, and these lead towards an initially immunonegative center. **c** Gradually, the soma of the affected pyramidal cell becomes AT8-immunopositive. Portions of the distalmost apical dendrite in the stratum moleculare show AT8-immunopositive spindle-shaped varicosities that are unique to the CA1 sector. **d** The dendritic connections to the soma become extremely thin and are finally lost. **e.** Pretangle tau converts to a fibrillar and argyrophilic NFT in the cell soma and to NTs in the dendrites. **f** After cell death, the cellular nucleus disappears. Both the steel wool-like fibrillar tau and the assemblies of lipofuscin granules can then be seen in the extracellular space (neuropil). **g**, **h** The NFTNTs are taken up by astrocytes [68, 69] and degraded, whereas the remaining neuronal lipofuscin granules are taken up by microglia. These pigmented glial cells eventually disappear from this location

In the present study, the initially involved hippocampal projection neurons of the prosubiculum, CA1, and CA2 gradually increased in number. Our findings suggest that this increase may be captured by a uniform mechanism: A single axon generated from cells of the outer entorhinal layers may split into two basic collaterals. Passing the prosubiculum, a basal branch would head downwards and contribute to the formation of the stratum oriens (Fig. 13). Close to its target cell, it would split into terminal branches. Each single branch would then terminate on its corresponding basal dendrite in a CA1 or CA2 pyramidal cell (Fig. 13). All of the dendrites of a single pyramidal cell would receive contacts from a single branch. None of the terminal collateral branches, however, would establish a connection to another cell in the vicinity. The second branch of the involved entorhinal collateral would follow the course of the perforant path and run within the stratum lacunosum (Fig. 13). From there, it would reach side branches of the apical dendrite of the same cell already contacted by the basal branch (Fig. 13). A concerted action by both branches then would result in the simultaneous appearance of abnormal pretangle tau in the distalmost portions of both the basal and apical dendrites of a specific nerve cell (identical shading in Table 4 implies that this simultaneous change by both branches may be assigned to the same time frame), suggesting a strongly focused point-to-point transmission from a single axon to a single neuron. Taken together, the distalmost dendritic segments of select hippocampal projection neurons initially bear the brunt of the AD-related tau pathology: A recapitulation of these consecutive alterations is presented schematically in Fig. 14.

The early tau pathology selectively and severely damages phylo- and ontogenetically late-emerging and late-maturing synapses along the distalmost dendrites [29] (Fig. 3a, b). Notably, these synapses are not essential for the most basic brain functions, i.e., survival [23]. Following the development of pathological changes in the prosubiculum/CA1/CA2 sectors, the tau pathology progresses into sectors CA3-CA4 (NFT stages II and III). The forceps-shaped accumulations of abnormal cellular processes in the stratum oriens and stratum lacunosum were seen to have thinned out considerably in CA3 and they were no longer visible in CA4 (Fig. 8). This raises questions about whether the subsequent course of the pathological process is still governed by entorhinal axons. Spines along affected dendrites normally remain devoid of abnormal tau. The thorny excrescences of CA3/CA4 projection cells, however, display a notable exception from this rule and show a pronounced and *early* involvement (NFT stage I in Fig. 10f, h). The fact that they were occasionally seen in isolation supports the view that they are the first cellular processes belonging to these cells to become affected. Nearly all of the excrescences of an individual mossy cell showed a faint but simultaneous AT8-positive immunoreaction. Among these were a few darkly stained and slightly larger spines. These spines could be interpreted to represent the actual contact zone for nerve cell-to-nerve cell transmission of tau seeds. The excrescences are known to receive their main afferents from the mossy fibers of dentate granular cells [51, 52, 118]. However, at this point in the disease process, granular cells of dentate fascia were largely uninvolved and, thus, unlikely to provide the seeds for transmission of tau to the excrescences. Therefore, we postulate the existence of an additional connection generated by a proportion of already-involved prosubicular/CA1 projection neurons that may, additionally and directly, contact the thorny excrescences of CA3 and CA4 (Fig. 13, *green connection*). Again, we speculate that the terminal boutons of a single axon would establish contact with all thorny excrescences of a single mossy cell and with no other immediately adjacent mossy cells.

The task that remains is to determine if the postulated connections based on the sequence of morphological changes described here actually exist (Fig. 13, *green connections*) and can be modelled experimentally, or to formulate alternative and sufficient explanations for the distinctive morphological features of the pathological process (Tables 3 and 4, Fig. 14). The realization of peculiar point-to-point connections between the axon of a single nerve cell to all of the dendrites of the next nerve cell in the neuronal chain, and this in the absence of contacts to adjacent neurons, is conspicuous and as yet unreported in the literature on point-to-point data transmission.

Finally, axonal projections of the multipolar CA4 mossy cells are known to terminate in the internal one-third of the dentate molecular layer [70, 106]. These projections also could possibly serve as a conduit for the disease process to the granular cells of the dentate fascia. Terminal branches of the perforant path, by contrast, fill from the outset of the pathological process the outer two-thirds of the molecular layer, but they are probably not responsible for the transmission of AD-related tau seeds, inasmuch as the involvement of the granular cells is a late event that occurs only after the development of abnormally altered axonal terminals in the inner third of the molecular layer, i.e., following the appearance of involved CA4 mossy cells. It remains enigmatic why the involved axon terminals of the perforant path in the outer two-thirds of the dentate (Fd) molecular layer refrain from transmitting tau seeds.

Larger amounts of abnormal tau occur comparatively late (from NFT stage III onwards) in the output region of the hippocampus, the subiculum. It receives information from CA1. CA1 pyramidal neurons become involved early and, for this reason, we currently have no explanation for the delayed involvement of the subiculum to tau inclusions. This problem could be resolved by future studies.

Loss of tau-positive neurons that occurs during the course of the disease documents itself in overview stainings, such as the pigment-Nissl (PN) method (Fig. 6, Suppl. Figure 3): After cell death, the neurofibrillary inclusions and intraneuronal deposits of lipofuscin granules lie in the extracellular space [11] (Fig. 6, Suppl. Fig. 3). Astrocytes are known to phagocytose ghost tangles [68, 69], whereas the neuronal lipofuscin granules can be taken up by microglia (Fig. 6c–f). The occurrence of neuronal lipofuscin in microglial cells is a useful marker of loss of pigment-laden nerve cells. Owing to their smaller size, the pigment-laden microglial cells can be distinguished from the comparatively voluminous pigment-laden hippocampal interneurons [12, 96]. A localized loss of pigment-laden pre-α cells in the lateral transentorhinal area, for instance, clearly marks the beginning of the long-lasting process of AD-related loss of nerve cells (Table 5, Fig. 6b).

**Table 5:**
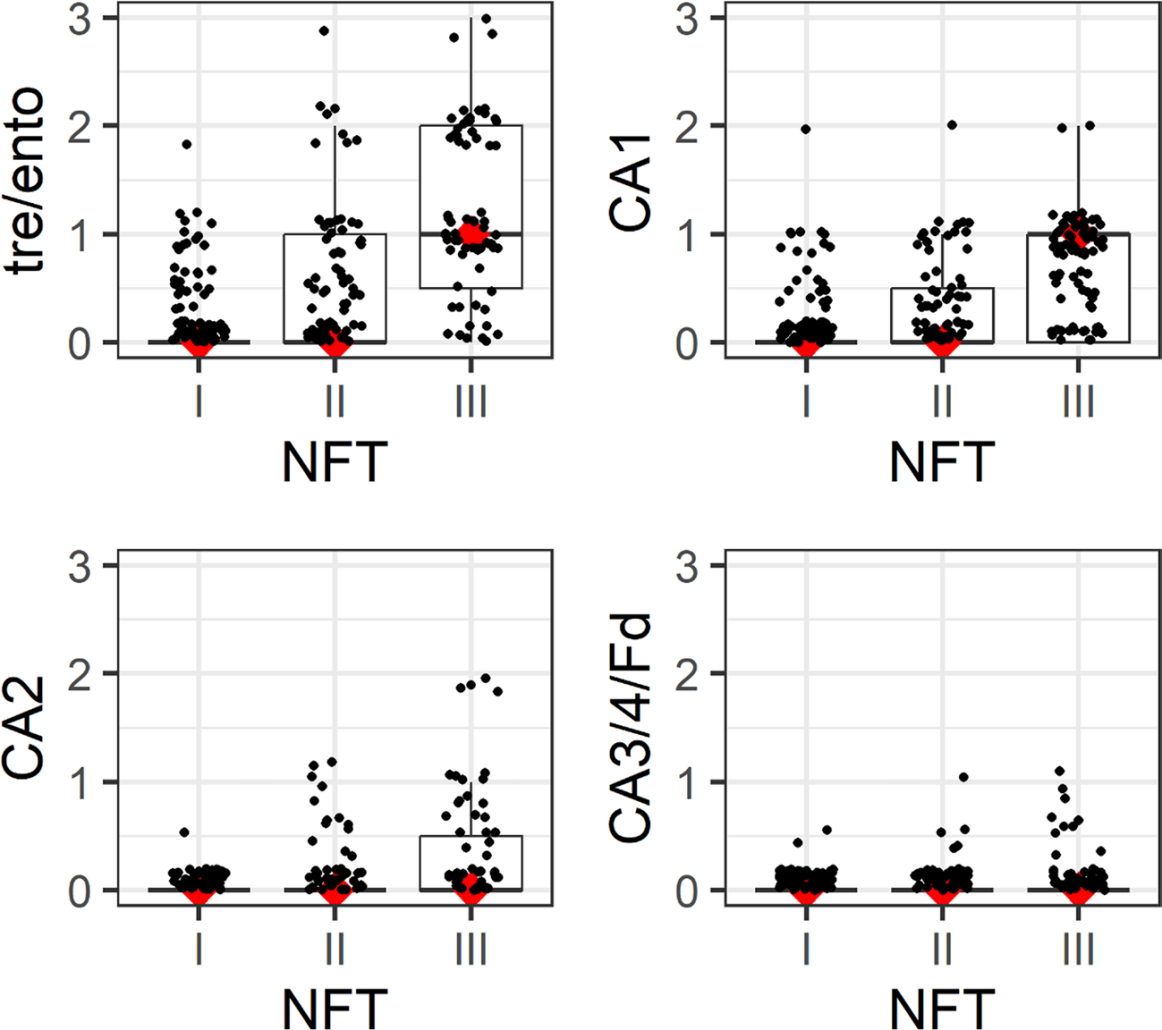
Neuronal loss according to NFT stages I–III

Neuronal loss in each region at each NFT stage was determined based on the presence of extraneuronal lipofuscin granules and pigment-containing microglia as markers of lost pigment-laden nerve cells. It was semiquantitatively assessed as 0 = none, 0.5 = subtle, 1.0 = mild, 2.0 = marked, and 3.0 = severe. Boxplots display median values (lines marked in *red*) and quartiles (upper and lower box limits); given the discontinuous rating scale these cannot always be distinguished. Based on these data, it can be seen that the difference between NFT stages is all the more pronounced the earlier the region in question is located in the cascade, i.e., that the progression of neuronal loss starts in tre/ento and continues through CA1, CA2, CA3, and CA4 to Fd. Whereas it becomes clear that higher ratings (up to 3) can be observed in the early-involved regions (tre/ento, CA1) and that these occur in NFT stage III, this effect, if at all, disappears in late-involved regions: CA2 tended to have higher ratings in NFT II and III, while in CA3/CA4/Fd there was no longer any difference between NFT I, II and II,I and the ratings there in general only ranged between 0 and 0.5

*tre/ento* transentorhinal/entorhinal regions, *CA1-CA4* cornu Ammonis (Ammon’s horn) sectors 1–4, *Fd* dentate fascia

Attempts have been made to separate the here-described early phase of abnormal tau from the AD-related process. Oligomeric Aβ is commonly considered as the driver and initiator of the development of abnormal tau in AD [61, 108; but see also 42, 49, 63, 75, 76, 80, 107]. According to this traditional view, brains that develop tau pathology in the absence of Aβ deposition is thought to represent a separate tauopathy (PART) that bears little or no relationship to the AD process. The postulated entity is described as a disease of elderly individuals, in whom tau inclusions remain restricted to medial portions of the temporal lobe [35, 36]. If, however, such cases are not on the AD continuum, they should commence in old age and show a numerical increase with increasing age. Our data, by contrast, show a Gaussian distribution of cases lacking Aβ plaques across nearly the entire life span (Table 1, Fig. 2). This contradicts the concept of an ‘age-related’ tauopathy that is separate from AD [44]. Moreover, the postulated entity—with advancing age—should hardly show fresh pretangle-containing nerve cells and, at the same time, increasing signs of neuronal loss. We did not find such cases in the present cohort, nor have they been reported in publications by other groups to date [129].

If the AD-related pathological process were indeed to be driven by the presence of Aβ, we would expect to find an abrupt appearance of abnormal tau in the temporal allocortex, i.e., a distribution of tau lesions corresponding to NFT stage III. Furthermore, such cases should almost exclusively display cells confined to the pretangle phase, and this is in the virtual absence of fibrillar NTs/NFTs or signs of nerve cell loss. However, of the N = 308 cases examined here, none displayed this profile. Thus, until the existence of such a pathological profile can be demonstrated, we think that the PART hypothesis is incorrect [38].

With increasing age, the Gaussian distribution of the cases shows a gradual decline (Fig. 2). This suggests that the early tau pathology is, with increasing age and step-by-step, replaced by later (higher) NFT stages. In other words, the early NFT stages are a necessary precondition for the later stages that result in the clinically detectable phase of AD [5, 22, 27, 37, 44, 49, 116].

### APOE status in early AD cases

Whereas the genotypes APOE ε2/ε2 and ε2/ε3 are associated with a reduced risk for late-onset AD (AD-related neuropathologic change, ADNC), and the age of AD onset increases with the number of APOE2 alleles, the ε4 allele of the apolipoprotein gene E is a major genetic risk factor for sporadic AD [7, 33, 34, 48, 125], TDP-43 proteinopathy, and Parkinson’s disease [99, 119, 126]. Individuals with the genotypes APOE ε3/ε4 or APOE *ε*4/ε4 have a two-fold to 12-fold increased risk for developing AD; the age-at-onset decreases with the number of an APOE ε4 alleles [33, 48, 84, 102]. A recent study regards the genotype APOE ε2/ε4 as a risk factor for developing more severe AD-related pathology (especially Aβ), mild cognitive impairment (MCI), and late-onset AD [97]. Notably, APOE ε4 may influence tau inclusions independently of Aβ deposition, and high tau seeding activity has been reported in APOE ε4/ε4 carriers [41, 56, 109].

Here, APOE genotyping was available for 188/308 individuals (Table 2). Our cohort included 2 ε2/ε2 carriers, 39 ε2/ε3, 50 ε2/ε4, 63 ε3/ε3, 31 ε3/ε4, and 3 ε4/ε4 carriers. Forty-one (21.81%) were ε2/ε2 and ε2/ε3 carriers and, therefore, were unlikely at higher risk for progressing to clinical AD. On the other hand, the presence of the human ε4 isoform is recognized as a major risk factor for sporadic AD. In our sample, 44.68% (84 of 188) individuals possessed an APOE ε4 allele (i.e., APOE ε2/ε4, ε3/ε4, ε4/ε4). We presume that all of them were at higher risk for developing clinical AD, including those at younger ages [7, 97] (Table 2). The percentages for our ε2/ε2 (1.06%) and ε4/ε4 (1.6%) carriers approximated those in the general population. The allele frequencies for **ε**2 and ε4 were elevated (overrepresented), and the ε3 frequency was lowered (underrepresented) in comparison to the general population (Table 2b, 2c, 2d). The mean ages for the various APOE genotypes hardly differed; thus, age did not play a role with regard to the genotypes in this cohort (Table 2e). In addition, when using the contingency table and chi-square statistics (chi-square test = 3.06, p = 0.38), no significant difference was found in the distribution of ε4 carriers across all age groups (20–100 years). However, the frequency of ε4 carriers in the age group < 50 years was lowest (31.3%) and highest (64%) in the age group between 50 and 60 years. Typically, in autopsy-based studies, the frequency of ε4 carriers seems to be highest, particularly in the age group 50–60 years given the selective mortality of ε4 carriers, inasmuch as the ε4 allele has detrimental effects on longevity, e.g., cardiovascular complications.

### Study strengths and limitations

A major strength of our study is that the histopathological workup for our early AD cases using the AT8 antibody originated from a single laboratory and, therefore, did not require harmonization of disparate institutional protocols. In addition, we excluded brains displaying comorbidities (mixed pathologies) that could have confounded our findings by influencing the presence and/or severity of the tau inclusions in our sample, or that could have impacted negatively on cognitive function [103]. A limitation that exists is inherent in cross-sectional studies, namely, any attempt to establish a sequence of processes or pathological changes based on isolated time points, e.g., at autopsy, results in hypotheses that are, by nature, not definitive. As such, the tau spreading hypothesis we proposed here and previously [25] requires testing and confirmation in large prospective longitudinal clinicopathological studies by independent laboratories.

In a recent study by the Giasson group [128], the authors, who used the antibody CP13 in addition to AT8 and a novel non-commercially tau antibody 3G12 (Ser208 that may also be a part of the classic AT8 epitope; see [87]), reported that the antibodies AT8, CP13, and 7F2 showed similar staining in human hippocampal sections for NFT, dystrophic neurites around neuritic plaques, and neuropil threads (see also their Figs. 5, 6 and 7 there comparing AT8 with CP13). AT8 has a high affinity for tau phosphorylated at S202 and T205, CP13 for tau phosphorylated at S202. Both antibodies recognize monomeric and assembled tau, with AT8 being easier to visualize because of the local concentration of phosphorylated tau. AT8, the antibody used here, is an exceptionally high-quality antibody. Thus, we have no reason to believe that CP13 shows an earlier stage of tau pathology than the current gold standard AT8 [67]. This would have to mean that S202 becomes phosphorylated before T205 in a tangle (neurofibrillary) tau and that the affinity of CP13 for phosphorylated tau is comparable to that of AT8.

A second study limitation is that APOE genotyping was only available for 188/308 (61%) cases and that the ε4 and ε2 alleles were overrepresented in our cohort compared to the general population [85, 88] (Table 2c). However, notably and in contrast to a previous study [90], the elevated frequency *ε*4 allele was associated here with definite PART.

Because we had no controls, the overrepresentation of the ε2/ε4 genotype cannot have resulted from a technical error; rather, the selection bias is anticipated and ‘normal’, as it were, inasmuch as we intentionally included 308 cases according to their underlying tau pathology: Cognitively unimpaired individuals of all ages were selected provided they had low NFT stages and lacked Aβ deposits. As such, it is not surprising that the ε2 allele frequency was elevated because cases with Aβ were excluded. The ε2/ε3 genotype frequency was also elevated, but this, too, would be typical for a cohort such as ours that lacked Aβ deposition [83, 101, 117]. Moreover, we excluded a very large number of co-occurring pathologies (e.g., hippocampal sclerosis, non-AD tauopathies, synucleinopathies, and ischemic stroke). Therefore, the available APOE genotypes are simply linked to this special population, which has not been studied in this manner previously and is most likely not representative of the average general population with mixed pathologies [85, 88]. In other words, the selection bias was intentional and attributable to our highly selective criteria. This accounts for the deviation in allele frequencies (Table 2).

## Conclusions

Here, we could show significant associations between early NFT stages I–III devoid of Aβ deposition and (**1**) anatomical regions as well as local specific cellular layers within each region displaying abnormal morphological changes (Tables 3, 4 both *p* < 0.001), (**2**) the here-proposed trend of tau progression within the hippocampal formation (Fig. 12, *p* < 0.001), and (**3**) tau-related neuronal loss (Table 5, *p* < 0.001 for transentorhinal/entorhinal regions, CA1/CA2; *p* = 0.003 for CA3/CA4/Fd). SYP-immunoreacitve presynapses were AT8-immunoreactive in early-stage AD (NFT stage I). Taken together, these findings are at least indirectly supportive of the anterograde spreading of tau inclusions within the allocortex, and they are supported by results from macaques, in which abnormal tau can arise independently of Aβ [37]. Our findings are incompatible with (**1**) the hypothesis that the presence of Aβ deposits is mandatory for the development of abnormal tau inclusions and neuronal loss in AD as well as (**2**) the concept that distinct neuropathological criteria should be applied to definite PART and AD. Thus, further inquiries and insights into the early tau phases during which Aβ deposition is lacking continue to be of fundamental significance for AD pathogenesis and are indispensable for the development of therapeutic interventions.

## Supplementary Information

Below is the link to the electronic supplementary material.

Supplementary file1 (DOCX 3741 kb)

## Data Availability

The data that support the findings of this study are obtainable from the corresponding author upon reasonable request.
