# Supplementary material for: Sequence and trajectory of early Alzheimer’s disease-related tau inclusions in the hippocampal formation of cases without amyloid-β deposits
Source: Acta Neuropathol. 2025 May 23;149(1):50. doi: 10.1007/s00401-025-02862-x (PMC12102137; doi:10.1007/s00401-025-02862-x)
Supplement: Supplementary file 1 — Supplementary file1 (DOCX 3741 kb) [file 401_2025_2862_MOESM1_ESM.docx]

**Supplementary Figures**

**Suppl. Figure 1. Ammon’s horn topography and anatomy.**


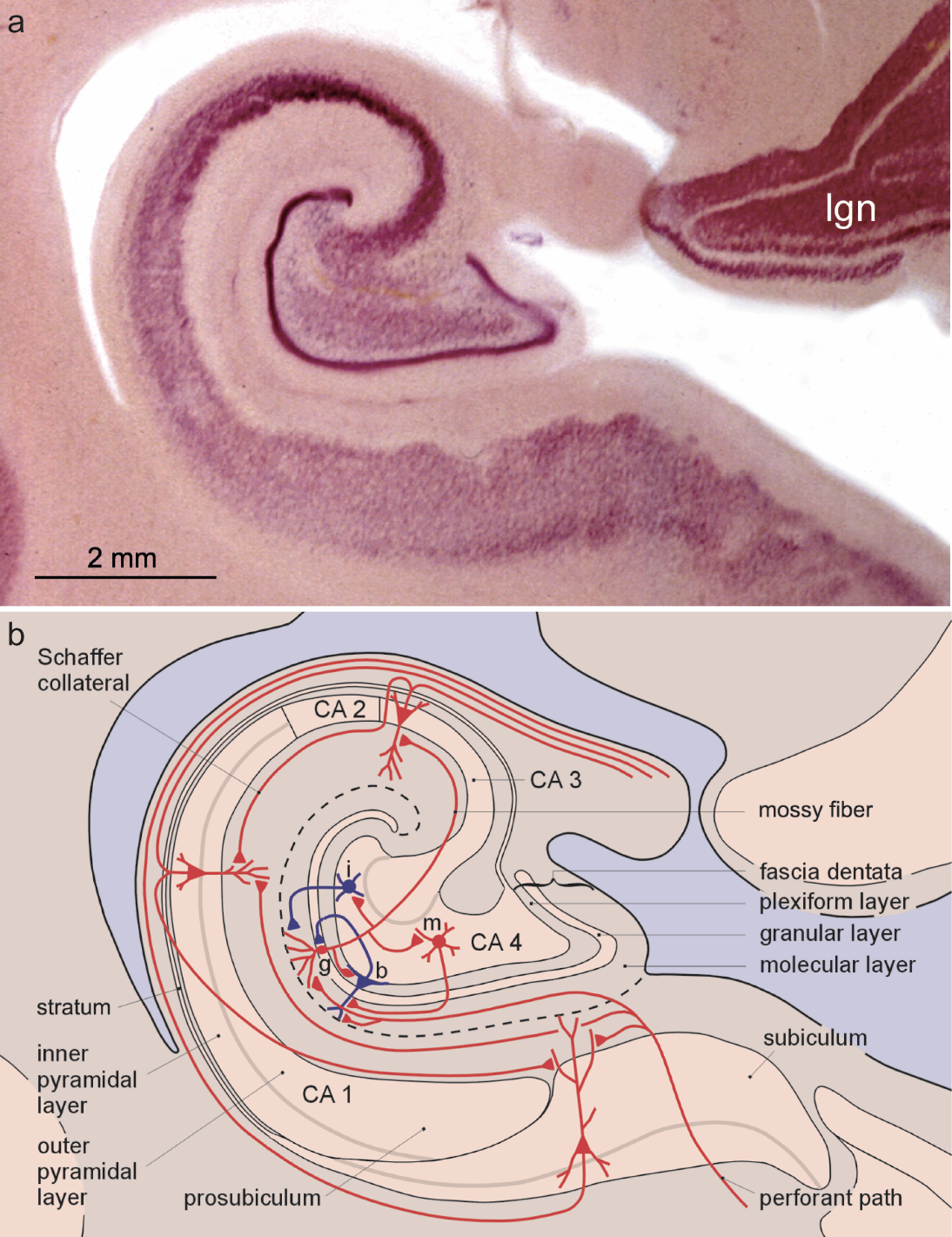


**a.** Aldehyde fuchsine-stained 100 µm section with Darrow red counterstaining (pigment-Nissl staining) for topographical orientation and recognition both of cellular laminae and different nerve cell types in the adult human brain. It also permits direct comparison of histological and topographical findings with neuroimaging [4]. The four sectors of Ammon’s horn (CA1-CA4) and the dentate fascia (Fd) belong to the temporal allocortex. **b.** CA1 (Sommer’s sector) is particularly extensive in the human brain [5] and differs from corresponding structures in the brains of non-primate mammalian species [1, 3]. Pyramidal cells of CA3 give rise to the Schaffer collaterals that terminate in the stratum oriens and stratum radiatum of CA1. CA1 pyramidal cells project to the subiculum; hippocampal output to the presubiculum, entorhinal cortex, amygdala, and ventral striatum originates mainly in the subiculum (pyramidal cells) [2, 3]. The grayish lines in CA1 and the subiculum separate the external from the internal pyramidal layer. **Abbreviations:** **lgn** – lateral geniculate nucleus, **b** – basket cell type (pyramidal form in the Fd, **g** – granule cell in the Fd, **m** – mossy cell in CA4).

**References**

1. Benavides-Piccione R, Regalando-Reyes M, Fernaud-Espinosa I, et al (2020) Differential structure of hippocampal CA1 pyramidal neurons in the human and mouse. Cereb Cortex 30:730-752. doi: [10.1093/cercor/bhz122.](https://doi.org/10.1093/cercor/bhz122)

2. Braak H, Braak E, Yilmazer D, Bohl J (1996) Functional anatomy of human hippocampal formation and related structures. J Child Neurol 11:265-275. doi: [10.1177/088307389601100402.](https://doi.org/10.1177/088307389601100402)

3. Insausti R, Amaral DG (2012) Hippocampal formation. In: Mai JK, Paxinos G, eds, The Human Nervous System, 3^rd^ ed. San Diego, Academic Press, 896-942.

4. Schröder H, de Vos RAI, Huggenberger S, Müller-Thomsen, Rozenmuller A, Hedayat F, Moser N (eds.) (2023) The Human Brainstem: Anatomy and Pathology. Springer, Cham (Switzerland). doi: <https://link.springer.com/book/10.1007/978-3-030-89980-6#about-this-book>.

5. Stephan H (1983) Evolutionary trends in limbic structures. Neurosci Behav Res 7:367-374. doi: [10.1016/0149-7634(83)90041-6.](https://doi.org/10.1016/0149-7634(83)90041-6)

**Suppl. Figure 2. Morphology of NFTs in the pre-α layer of the entorhinal region and CA1.**


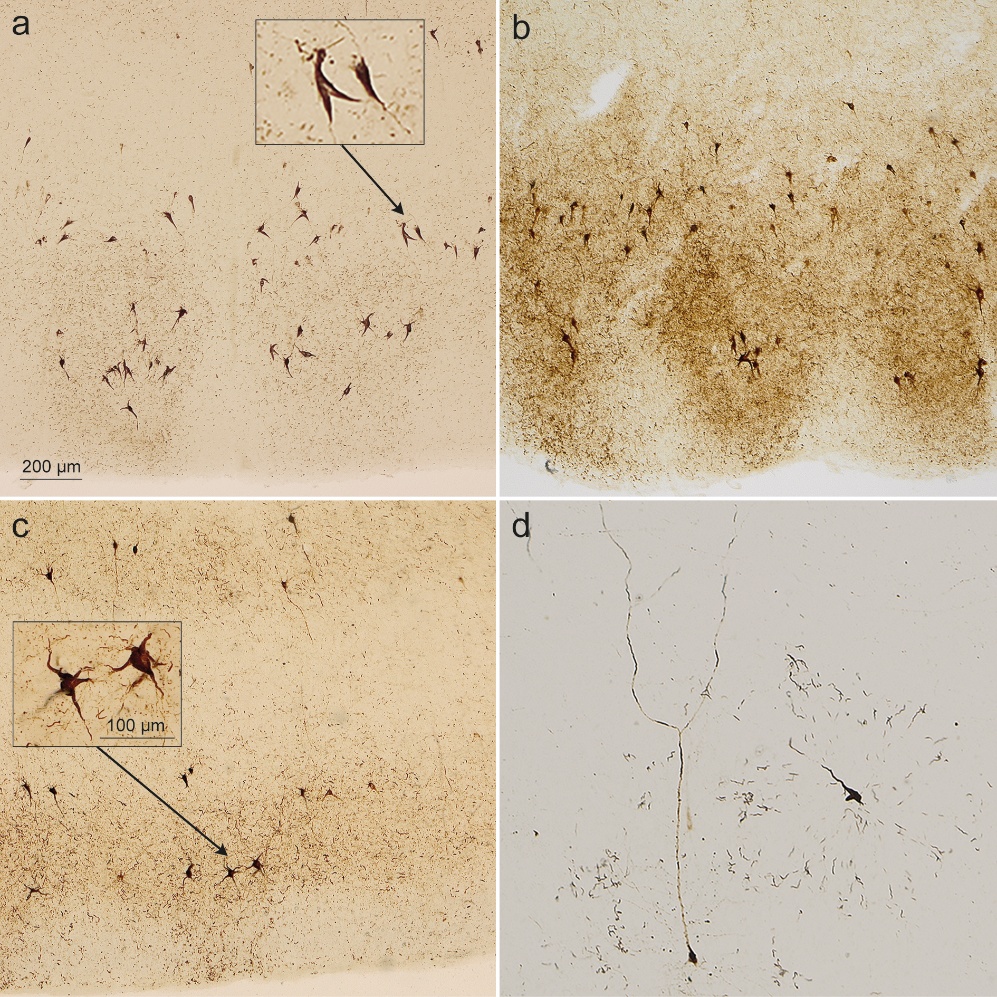


**a-c.** Most NFTs in pyramidal cells of the transentorhinal / entorhinal (*framed area* in **a**), CA1 regions (**d**), and subiculum are flame-shaped and elongated, whereas are star-shaped (*framed area* in **c**). By contrast, globose (globular) NFTs are found in CA2-CA4 and in the dentate fascia (see Fig. 10e above). **a**. Entorhinal region, male 78 years of age, NFT II, APOE ԑ2ԑ4. **b.** Entorhinal region, female 77 years of age, NFT III, APOE ԑ3ԑ3. The cellular island of the pre-α layer also displays a thick network of NTs. **c.** Entorhinal region, male 57 years of age, NFT II, APOE ԑ3ԑ3. **d.** CA1, male 62 years of age, NFT I, APOE ԑ3ԑ3. 100 µm sections. AT8-immunohistochemistry. Scale bar in **a** applies to **b-d**. Bar in *framed area* of **c** also applies to *framed area* in **a**.

**Suppl. Figure 3. Extraneuronal lipofuscin pigment in CA2 and CA4 shown in staining with aldehyde fuchsine and Darrow red.**
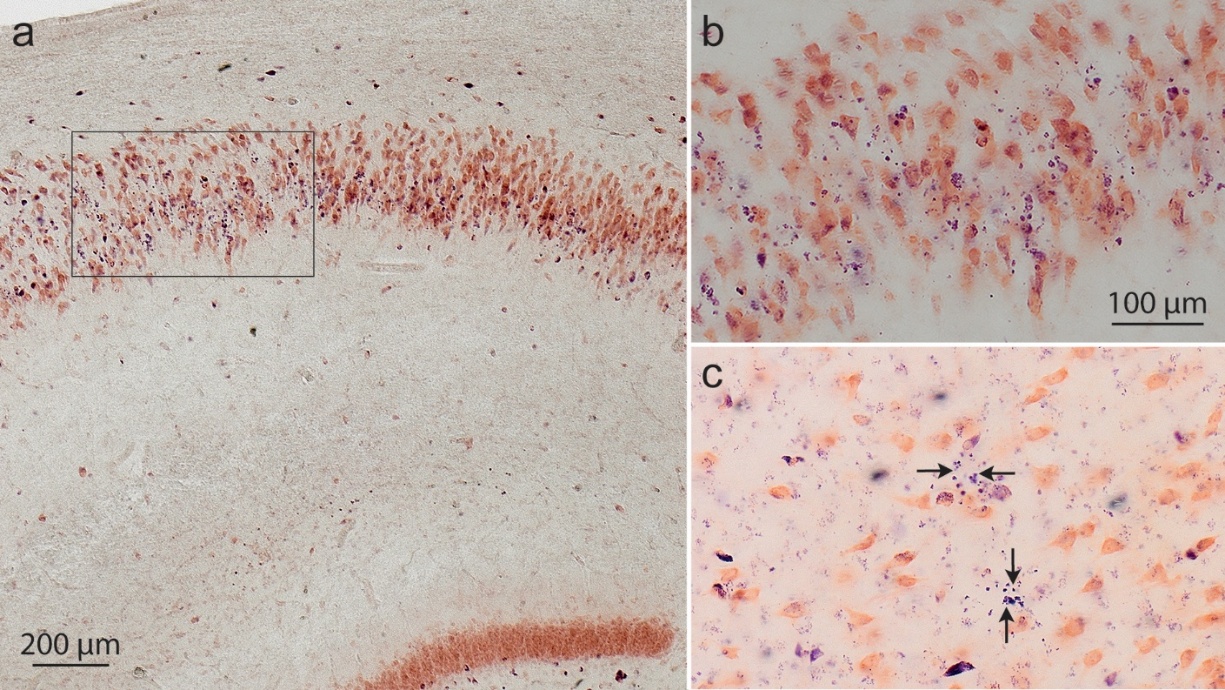


**a, b.** Overview and detail of the deep pyramidal layer and the superficial pyramidal layer in sector CA2. *Framed area* in **a** is shown in greater detail in **b**. 100 µm section, male 91 years of age, NFT stage III, APOE unavailable. **c.** Local accumulations of extraneuronal pigment granules lying free in the neuropil of the CA4 sector (*arrows*). 100 µm section, male 72 years of age, NFT III, APOE ԑ3/ԑ4. The gradual progress of neuronal loss can be approximately followed throughout the various regions affected during the AD-related pathological process. Bar in **b** also applies to **c**.
